# Supplementary material for: Persistent trade-offs balance competition and colonization across centuries
Source: Proc Natl Acad Sci U S A. 2026 Jun 2;123(23):e2534310123. doi: 10.1073/pnas.2534310123 (PMC13250502; doi:10.1073/pnas.2534310123)
Supplement: Supplementary file 1 — Appendix 01 (PDF) [file pnas.2534310123.sapp.pdf]

PNAS

Supporting Information for Persistent trade-offs balance competition and colonization across centuries

Talia Backman*[1], Jiajun Cui*[2], Emma Caullireau*[1], Ella Bleak[1], Ilja Bezrukov[3], Patricia Girardi[1], Aubrey Hawks[1], Jesse R. Lasky[4,5], Sergio M. Latorre[2], Joel M. Erberich[6], Lua Lopez[4,7], Manuela Neumann[3], Allison M. Perkins[1], Efthymia Symeonidi[1], Parastoo Azadi[8], Martin P. Horvath[1], Artur Muszyński[8], Patricia L. M. Lang[9], Talia L. Karasov*[1]*✝, Hernán A. Burbano*[2]✝

Affiliations:
[1]School of Biological Sciences, University of Utah, Salt Lake City, UT 84112, USA.
[2]Centre for Life's Origins and Evolution, Department of Genetics, Evolution and Environment, University College London, London WC1E 6BT, UK
[3]Department of Molecular Biology, Max Planck Institute for Biology Tübingen, Tübingen 72076, Germany
[4]Department of Biology, Pennsylvania State University, University Park, PA 16802, USA
[5]PAC Herbarium, Pennsylvania State University, University Park, PA 16802, USA
[6]Department of Biology, Stanford University, Stanford, CA, USA
[7]Department of Biology, California State University, San Bernardino, California, USA
[8]Complex Carbohydrate Research Center, University of Georgia, Athens, GA 30602, USA
[9]Department of Plant and Microbial Biology, University of California Berkeley, Berkeley, CA 94720, USA

*Equal contribution
✝Corresponding author

Contact:

Talia L. Karasov (t.karasov@utah.edu) and Hernán A. Burbano (h.burbano@ucl.ac.uk)

This PDF file includes:

Supporting Text
Figures S1 to S19
Tables S1 to S8
SI References

Supporting Information Text

Material and methods

Bacterial strains, plasmids and growth conditions

The strains used in this study are from Karasov et al. 2018 (1) (ENA: PRJEB24450). Bacteria were grown on Luria-Bertani broth agar (LB agar) and in Luria-Bertani (LB) medium at 28 °C for *Pseudomonas spp.* strains and 37 °C for *Escherichia coli* strains. All liquid cultures were incubated with shaking at 180 rpm. Antibiotics were used when appropriate at the following concentrations: 50, 100, and 10 µg/ml for kanamycin (Km), nitrofurantoin (NFN), and tetracycline (Tc), respectively. All strains were stored at −80 °C in 30% glycerol [v/v]. For plasmid preparation, *E. coli* transformants were cultured in LB. For the selection of the second *P. viridiflava* homologous recombination event, LB supplemented with sucrose (10 g/L tryptone, 5 g/L yeast extract, 10% (W/V) filtered sucrose, 15 g/L agar) plates were used.

Tailocin extraction and partial purification

Methods were adapted from Backman et al. 2024 (8). Overnight cultures were back-diluted into 50 ml of fresh LB to extract and isolate tailocins from the *P. viridiflava* strains. When cultures reached an exponential growth phase (an optical density at 600 nm (OD_{600}) of 0.4 to 0.6), 5 µg/ml MMC (Selleck, catalog no. S8146) was added to induce the bacterial SOS response and tailocin induction. The cultures were then incubated at 28 °C for a minimum of 18 hours and then centrifuged at 4 °C for 1 hour at 1,400 g to pellet cell debris. The supernatants were sterilized by filtration with 0.2-µm cellulose acetate filters. To precipitate tailocins, 40% (w/v) of ammonium sulfate was slowly added to the filter-sterilized tailocin lysate while stirring on ice. The lysate was left stirring on ice for at least 18 hours. After 18 hours at 4 °C, the ammonium sulfate was pelleted by centrifugation at 2,090 g for 2 hours at 4 °C. The supernatant was discarded, and the pellet was resuspended in 500 µl of cold P-buffer (100 mM NaCl, 8 mM $MgSO_4$, 50 mM Tris-HCl, pH 7.5) and stored at 4 °C. Tailocin lysates were used fresh or for up to 2 weeks and stored at 4 °C.

Testing bacterial sensitivity to tailocins with spot test phenotypic assays

Methods were adapted from Backman et al. 2024 (8). Briefly, to test the sensitivity of the different bacterial strains to the tailocins, soft agar assays were performed using an adaptation of a protocol from Vacheron et al. (2). Overnight cultures (750 µl) of each strain were mixed with 25 ml of LB soft agar (0.8%), and the mixture was poured into a square plate and left to harden. Then, aliquots of 3 µl of concentrated tailocins suspension were applied to the agar, along with a control of P-buffer alone and noninduced cultures in serial dilutions. The plates were incubated overnight at 28 °C. Bacterial sensitivity or resistance to the tailocins was assessed after 24 hours. No plaques were observed when testing serially diluted tailocins, suggesting that the killing agent is nonreplicative and not a phage. The p25.A12 tailocin can kill p25.C2 WT, and was used for mutant spot test phenotypic

assays. There were three biological replicates per condition and eight serial dilutions.

Plant infections of *Pseudomonas* natural isolates

A. thaliana genotypes Eyach 1.5-2 (15-2) and Col-0 were grown axenically and infected with single *Pseudomonas* strains as described in (1). In brief, seeds were sterilized by overnight incubation at −80 °C, followed by 4 hours of bleach treatment at room temperature (seeds in an open 2 ml tube in a desiccator containing a beaker with 40 ml Chlorox and 1 ml HCl (32%)). Seeds were then stratified for three days at 4 °C in the dark on ½ MS media. Plants were grown in 3-4 ml ½ MS medium in six-well plates in long-day (16 hours) at 23 °C. 12-14 days after stratification, plants were infected with single bacterial strains.

Pseudomonas overnight cultures were diluted at 1:10 in 5 ml of selective media and grown for an additional three hours. Bacteria were then centrifuged at 3,500 g and the pellets diluted to an optical density (OD_{600}) of 0.01 in 10 mM $MgSO_4$. A hundred microliters of this bacterial suspension were used to drip-inoculate plants, distributing the volume over the whole rosette. Plants were infected with 10 mM $MgSO_4$ as a control. The plates were sealed with parafilm and then returned to the growth chamber. Seven days after infection, pictures of rosettes were taken for green pixel quantification. At least three biological replicates were performed per infection.

Plant health quantification with green pixels

For plant health quantification, plates were photographed seven days post-infection, with a tripod-mounted Canon PowerShot G12 digital camera. Individual plants were extracted from whole-plate images as described in (3). The number of green pixels was determined for each plant and used as a proxy for plant fresh mass. The segmentation of the plant from the background was performed by applying thresholds in LAB color space, followed by a series of morphological operations to remove noise and non-plant objects. Finally, a GrabCut-based postprocessing was applied, and CSV files with plant IDs and green pixel counts were created. The workflow was implemented in Python 3.6 and bash using OpenCV 3.1.0 and scikit-image 0.13.0 for image processing operations (3).

Competitive mutant fitness assays *in planta*

Columbia-0 (Col-0) and Eyach 1.5-2 WT *A. thaliana* plants were grown under long-day conditions (16 hours light, 8 hours dark) in an AR41L3 Percival detector with 60% intensity of the SciWhite LED lights. Seedlings were grown in 24-well plates (Greiner Bio-One, catalog no. 6621665). Thirteen-day-old seedlings were used for the infections. Plants were infected with bacterial suspension of the *P. viridiflava* p25.C2 saturation mutagenesis library at an OD_{600} of 0.02. Each plant was infected with 200 µL of bacterial suspension. Each biological sample contained two infected plants. Three days after infection, samples were collected in 2-ml deep-well plates and snap-frozen. Material was ground using two 5-mm glass beads and DNA was extracted using the Puregene extraction kit (QIAGEN Gmbh, Hilden, Germany).

RB-TnSeq and analysis of RB-TnSeq data

Methods were adapted from (8). Briefly, genomic DNA was extracted and barcode PCR was performed as described in (4). DNA extractions were quantified with NanoDrop 1000 (ThermoFisher Scientific). Barcode sequence data were obtained by multiplexing on a partial NovaSeq X plus lane (Illumina, San Diego, United States) at Novogene (Novogene Corporation Inc., Sacramento, United States). Fitness data were calculated and analyzed from these reads with the DESeq2 R package (5), and scripts can be found on our GitHub page.

Construction of the p25.C2 *Pseudomonas* gene deletions

The p25.C2 mutant strains (Δ*OBC*, Δ*wfgD*, Δ*rmlC_1*, Δ*tagG_2*, Δ*tagH_2,* Δ*epsE_4*, and Δ*spsA*) were obtained using the Gateway cloning system with the donor vector pDONR1K18ms (Addgene plasmid no. 72644) and the destination vector pDEST2T18ms (Addgene plasmid no. 72647). Briefly, attB-flanked upstream and downstream gene fragments were ordered from TWIST (6) (**Table S5**). BP and LR reactions were conducted in the one-tube format using the attB-flanked fragments. The resulting expression clone was transformed into competent *E. coli* DH5α and then introduced into recipient cells (*P. viridiflava* p25.C2) by conjugation. Recombinant cells (merodiploids) were selected for with the antibiotic resistance conferred by the expression clone and verified with PCR and gel electrophoresis. Positive colonies were purified by streaking onto new plates. Purified merodiploids were grown overnight without the antibiotic conferred by the expression clone to allow for another recombination event and then plated on 10% D-sucrose for *sacB* counterselection. Colonies were examined by PCR and verified by DNA sequencing (GeneWiz). The plasmids for *tagH_2* and *spsA* were ordered directly from TWIST.

LPS isolation and purification

Bacteria were grown overnight at 28 °C under vigorous agitation (200 rpm). The suspensions were centrifuged, and the pellets were washed three times in PBS. Wet cell pastes were stored at -70 °C until LPS extraction. The cells were uniformly suspended in deionized water (ratio 1:5 w/v) and the suspensions were brought up to 68 °C with gentle stirring. The extraction was performed by adding an equal volume of preheated to 68 °C 90% liquefied phenol (w/w) and stirring for 20 min at 68 °C, following the Westphal procedure (7) and as previously described (8). Nucleic acids and proteins were removed by treatment with Benzonase (15 U/ml of LPS stock for 18 h, 37 °C, in the 50 mM $MgCl_2$ and 20 mM NaOAc buffer at the pH 7.6), followed by Proteinase K (0.66U/ ml, 18 h, 37 °C), and dialysis (12-14,000 MWCO) at 4 °C against several exchanges of deionized water. Dialyzed fractions were freeze-dried, dissolved, and precipitated three times in cold (-20 °C) 90% EtOH. LPS pellets were freeze-dried again and dissolved in deionized water, and additionally purified by ultracentrifugation at 100,000 g at 4 °C for 16 h. The enzymatic purification and the ultracentrifugation were repeated twice to obtain ultrapure LPS, which was verified by chemical analysis.

Analysis of pure LPS by DOC-PAGE

One microgram of purified LPS samples in Laemmli buffer were resolved in 0.75mm-Polyacrylamide gel electrophoresis (PAGE, 4% stacking gel and 18% resolving gel) in the presence of deoxycholic acid (DOC) detergent (9) for 1 h at 400 V, 30 mA. *Salmonella enterica* ser. *typhimurium* S-type LPS (O-antigen producing strain) was used as a standard. The PAGE was fixed overnight in an aqueous solution of 40% ethanol and 5% acetic acid, and the LPSs were visualized using a silver stain reagent kit (Bio-Rad, CA, USA) after oxidation with sodium periodate.

Chemical analysis of pure LPS

Analysis of the glycosyl residues constituting LPS was achieved by derivatizing the samples to O-trimethylsilyl (TMS) methyl glycosides. Briefly, samples were methanolyzed with 1 M HCl-methanol at 80 °C for 18 h, re-N-acetylated at 100 °C for 1 h, and O-trimethylsilylated with Tri-Sil reagent (Thermo-Fisher) at 80 °C for 30 min. Each sample was supplemented with an internal standard of myo-inositol (10, 11). GC-MS analysis of the LPS derivatives was performed on an Agilent AT 7890A GC system interfaced to a 5975B MSD using an Equity-1 (Supelco) fused silica capillary column (30 m length x 0.25 mm ID x 0.25 µm film thickness). The temperature gradient was 80 °C for 2 min, then increased to 140 °C at 20 °C/min with a 2-min hold, followed by an increase to 200 °C at 2 °C/min. Finally, the temperature was increased to 250 °C at 30 °C/min with a 5-min hold. The data were processed using Agilent ChemStation.

Plant infection by flood inoculation

To evaluate the growth of p25.C2 WT and O-antigen mutants *in planta*, Columbia-0 and Eyach 1.5-2 WT *A. thaliana* plants were grown under long-day conditions (16 hours light, 8 hours dark) in an AR41L3 Percival detector with 60% intensity of the SciWhite LED lights. Seedlings were grown in 24-well plates (Greiner Bio-One, catalog no. 6621665). Thirteen-day-old seedlings were used for the infections. Plants were infected with bacterial suspension of the *P. viridiflava* strain p25.C2, O-antigen mutants, or buffer (10 mM $MgSO_4$). All bacterial strains were tagged with luciferase (3). Bacteria were grown overnight and diluted 1:10 on the day of the infection. Cells were grown for another 3 hours and then collected and resuspended in 10 mM $MgSO_4$ to a final OD_{600} of 0.01. The plants were flood-inoculated for 1 min in a randomized manner with 1 ml of treatment (WT, mutant, or buffer).

Bacterial fitness evaluation

After flood-inoculation, plants were grown for 7 days and then collected. The collected plants were ground in 1 ml of 10 mM $MgSO_4$ using the Qiagen tissue lyser II, and 200 µl of the suspension was used to measure luciferase activity with a microplate plate reader (Spark®, TECAN, Switzerland). Plants infected with $MgSO_4$ were used as controls. In total, 24 plants were used for each strain or buffer. Plants were blindly scored as healthy (only green, healthy tissue), diseased (chlorosis phenotype, may have some white tissue), or dead (necrosis phenotype, full white tissue).

To assess differences in bacterial growth across strains, we used linear mixed-effects models with batch as a random effect and strain as a fixed effect, using the lmer() function in the lme4 R package. Pairwise comparisons between WT and mutant strains were performed using the emmeans() function from the emmeans package. P-values were adjusted for multiple comparisons using the False Discovery Rate (FDR) method, accounting for all pairwise contrasts among strains. This approach enabled us to test for significant differences in colonization while controlling for inflated Type I error rates resulting from multiple tests.

Oxidative stress tolerance assays

Methods were adapted from Binesse et al., 2015 (12). To assess sensitivity to reactive oxygen species, we measured growth of WT and O-antigen mutants across a range of hydrogen peroxide (H_2O_2) concentrations using a microplate growth assay. Overnight cultures were diluted into fresh medium and inoculated into 96-well plates containing 0–10 mM H_2O_2 in technical replicates. Optical density (OD_{600}) was recorded every 15 minutes for 24 hours at 28 °C using a microplate reader (Spark®, TECAN, Switzerland). Four biological replicates were done.

For each plate (replicate), raw OD_{600} time series were converted to growth area (AUC) using the trapezoidal rule over 0-24 h. AUC values for each strain were normalized to their respective 0 mM control to yield AUC_{rel0}. To account for plate-to-plate variation in baseline H_2O_2 potency, we estimated a per-plate WT minimum inhibitory concentration (MIC), defined as the first concentration where mean AUC_{rel0} dropped below 0.1. All H_2O_2 concentrations on that plate were then scaled by the corresponding WT MIC to generate a standardized dose axis (H_2O_2_scaled).

To test for differences in dose-response profiles between WT and O-antigen mutants, we fitted a linear mixed-effects model of log-transformed AUC values, log(AUC_{rel0}) = strain x H_2O_2_scaled + (1 | replicate), using the lmerTest package in R. Estimated marginal means (EMMs) and pairwise contrasts versus WT were computed with the emmeans package on the response scale.

To directly compare MIC values, we also estimated a strain-specific MIC for each replicate using linear interpolation of AUC_{rel0} versus H_2O_2 concentration. A second mixed model of log-transformed MIC values, log(MIC) = strain + (1 | replicate), was used to test for differences in inhibitory thresholds.

Disk diffusion assay

Bacteria were grown for 16 h at 28 °C with vigorous agitation (200 rpm). Suspensions were back-diluted 1:10 in 5 ml of selective liquid medium and grown for an additional 3 h. Suspensions were adjusted to OD_{600} = 0.6 and streaked as a thin, uniform layer onto selective solid medium using sterile cotton swabs. Sterile 6 mm filter paper disks were saturated with 20 µL of antimicrobial compound solutions (polymyxin B or colistin sulfate) prepared at concentrations ranging from 0 to 500 µg/ml. Once dried, the disks were placed on the bacterial layer, ensuring sufficient distance between them to prevent overlap of inhibition zones. Inhibition radii were measured after overnight static incubation at 28 °C. The absence of

significant differences among strains at each compound concentration was assessed using either one-way ANOVA or Kruskal–Wallis tests, as appropriate.

Aggregation formation assay

Aggregation formation was assessed using a 96-well plate growth assay. Overnight cultures of each strain were grown in LB medium at 28 °C with shaking. The following day, cultures were back-diluted 1:10 in fresh LB and grown to an OD_{600} of approximately 0.5. Each culture was then diluted 1:40, and 200 µL of the resulting suspension was added to wells of a sterile, flat-bottom polystyrene 96-well plate (≥10 technical replicates per strain). Wells containing 200 µL of sterile LB served as negative controls. Plates were incubated statically at 28 °C for 48 h. Biofilm formation assays were done in three biological replicates.

After incubation, wells were visually blindly scored as aggregation (visible surface-associated growth), no aggregation (turbid growth without surface attachment), or clear (no growth). For statistical analyses, aggregation presence/absence was coded as a binary trait. Proportional differences among strains were first tested with a Pearson's chi-square test, followed by pairwise Fisher's exact tests comparing each mutant to the WT strain (p25.C2). Resulting p-values were adjusted for multiple testing using the Benjamini-Hochberg procedure.

Immune gene expression assessment

Following flood-inoculation, rosettes were collected at 0, 2, 6, 10, 24, 48 and 72 hours post-infection. For each treatment modality at each time point, three individual plants were pooled to form one biological replicate, and this was repeated 3 times to obtain the biological replicates. Samples were snap frozen immediately after collection and stored at -80 °C until RNA extraction. Frozen material was ground using the Qiagen tissue lyser II and total RNA was extracted with TRIzol™ reagent (#15596026; Invitrogen, Thermo Fisher Scientific, MA, USA). RNA concentration, purity and integrity were verified spectrophotometrically and by gel electrophoresis. One microgram of RNA was treated with 1 unit of DNase I (#EN052; Thermo Fisher Scientific, MA, USA) and reverse-transcribed using 50 ng of random hexamers and 200 units of SuperScript™ IV (#18090200; Thermo Fisher Scientific, MA, USA). The resulting cDNA was treated with 2 units of *E. coli* RNase H (#18090200; Thermo Fisher Scientific, MA, USA) and diluted 1:20 to serve as a template for *PP2A* amplification (27 cycles), confirming successful cDNA synthesis. Samples were diluted 1:50 and used as templates for qPCR reactions performed with Luna® Universal qPCR Master Mix (New England Biolabs, MA, USA), using 20% of cDNA per reaction. All samples from one biological replicate were run on a single 384-well qPCR plate, with three technical replicates per target gene. Amplification was performed on a CFX Opus 384 Real-time PCR system (**Table S6**) and melt curves quality was assessed using the Maestro CX software (Bio-Rad, CA, USA). Amplification data were analyzed in RStudio using the chipPCR and qpcR packages. PCR efficiencies were calculated for each individual well and incorporated into the computation of mean normalized expression (13, 14). Expression of *FRK1*, *WRKY29*, *NHL10*, *PAD3*, *PDF1.2*, and

PR1 was quantified over time relative to the housekeeping gene *PP2A*, and normalized to the mock-treated samples at each time point. For each target gene, significant differences between the WT and O-antigen mutants at each time point were assessed using one-way ANOVA or Kruskal–Wallis tests, as appropriate.

_K_-mer–based analysis of tailocin genomic similarity across the _Pseudomonas syringae_ species complex

Publicly available *Pseudomonas syringae* genomes were downloaded from Pseudomonas.com and filtered for assembly quality (assembly length 5.5–7.5 Mb, <1,000 contigs, and N50 >100 kb). To reduce redundancy, genomes were clustered at 99% average nucleotide identity (ANI), and one representative genome with the best assembly metrics was selected per cluster. This resulted in a dataset of 126 *Pseudomonas syringae* genomes. For each genome, the tailocin cluster, flanked by the *trpE* and *trpG* genes, was extracted based on NCBI's GenBank annotations (GenBank files downloaded with NCBI datasets API) and validated with *Pharokka* (15) and *PHOLD* (16). The location of the *HTF* genes was inferred based on synteny within the tailocin cluster. For the 53 *Pseudomonas viridiflava* ATUE5 assemblies, the tailocin cluster was likewise defined as the region between the *trpE* and *trpG* genes. Using the strain p25.C2 as the reference, the tailocin cluster in each strain was identified using Minimap2 v2.28 (17). When tailocin genes spanned multiple contigs, matched segments were concatenated to reconstruct the full sequence. *HTF* sequences were extracted and open reading frames were manually inspected. Tailocin genomic similarity between 53 *Pseudomonas viridiflava* ATUE5 genomes and 126 *Pseudomonas syringae* genomes was then assessed using panKmer 0.20.4, a *k*-mer based analysis tool (18). For each species, all possible 31-nucleotide *k*-mers were generated from nucleotide sequences of both the whole tailocin gene cluster excluding the tail fiber gene (*HTF*) and for the *HTF* alone. Subsequently, pairwise similarities were computed as Jaccard distances from panKmer adjacency matrices. Afterwards, Principal coordinates analysis (PCoA) was performed on the resulting distance matrices using the PCoA function from the scikit-bio Python package (19). Unrooted neighbour-joining trees were reconstructed from the same matrices using the DistanceTreeConstructor function from Biopython (20). Tail fibers were classified as Type1, Type2, or Type3 by BLASTp against 10 reference proteins representative of all three types (21). BLASTp hits were ranked by bitscore and sequences that did not pass an e-value threshold of 10^{-5} were excluded. Phylotypes were assigned to *P. syringae* strains by ANI comparison against reference strains from each phylotype (22). Genomes with ≥95% ANI to a given reference were assigned to that phylogroup (using the highest-scoring reference where multiple references exceeded the threshold), whereas those below 95% ANI with all references remained unassigned.

DNA extraction, library preparation and sequencing of _A. thaliana_ herbarium specimens

Herbarium specimens of *Arabidopsis thaliana* spanning almost two centuries (between 1817 and 2015) were obtained from seven European institutions: Staatliches Museum für Naturkunde Stuttgart (Stuttgart, Germany), the Herbarium

Tubingense (Tübingen, Germany), Lund University Botanical Museum (Lund, Sweden), the herbarium at the Biological Museum Oskarshamn (Oskarshamn, Sweden), the herbarium at Real Jardín Botánico (Madrid, Spain), the Herbario de Málaga (Malaga, Spain) and The Natural History Museum's herbarium (London, United Kingdom). Small tissue samples (~0.7 cm^2) were collected for DNA extraction and sequencing, with care taken to minimize visible damage. Each sample specimen was labelled with collection details and internal identifiers, and DNA extraction followed established protocols (23).

DNA from herbarium specimens was extracted and processed into single-stranded Illumina libraries at the UCSC Ancient and Degraded DNA Processing Center following Kapp et al. (24) with suggested modification from Nguyen et al. (25). Strict contamination control and cleanroom procedures were implemented. Libraries were initially shallow-sequenced on an Illumina NextSeq 550 to assess DNA quality and plant endogenous DNA content, then sequenced on a NovaSeq X Plus (2x150 bp), while adjusting per-sample concentration in the DNA pools to finally achieve an average depth around 9X for the host *A. thaliana* genome of each sample. All sequencing reads have been deposited in the European Nucleotide Archive (ENA) under project ID: PRJEB98841.

Historical reads processing, mapping and authentication

We identified *Pseudomonas*-derived reads in 49 *A. thaliana* herbarium specimens collected across Europe between 1817 and 2015, comprising two previously published datasets (26, 27) and 31 newly sequenced herbarium samples from this study (**Table S7**). Before read mapping, we used AdapterRemoval v2.3.3 (28) to trim adapters, remove low-quality bases, and collapse overlapping paired-end reads. Host-derived reads were removed by mapping all merged reads to the *A. thaliana* TAIR10 reference genome (29) using BWA aln v0.7.17 (30), with the seed disabled to improve alignment of damaged historical reads (23). For each library, reads that could not be merged due to long insert sizes were mapped independently as forward and reverse reads. Subsequently, merged and unmerged reads were combined into a single BAM file per library. The remaining unmapped reads were then mapped to the *P. viridiflava* ATUE5 p25.C2 reference genome (1) using the same approach. PCR duplicates were removed, and mapped reads were filtered for mapping quality ≥ 20 using samtools v1.11 (31). To authenticate the historical nature of both *A. thaliana*- and *Pseudomonas*-mapped reads, we used mapDamage2 v2.20 (32), which generated misincorporation patterns and length distributions typical of ancient DNA.

Phylogenetic placement of historical *Pseudomonas* in the context of modern diversity

To determine the phylogenetic placement of the 49 historical *Pseudomonas* strains in the context of modern diversity, we used a set of 83 contemporary genomes representing the known diversity of *Pseudomonas* segregating in wild populations. Single Nucleotide Polymorphisms (SNPs) were identified using bcftools v1.11 with the parameter -ploidy 1 (31), filtered for quality (QUAL >= 20), merged across samples, and restricted to biallelic sites with no missing data. A maximum-

likelihood tree was constructed from 3,095 biallelic SNPs detected in at least 95% of isolates using IQ-TREE v2.1.4 (33) applying TVM+F+ASC+R3 as the best substitution model (34). Historical strains clustering within the modern ATUE5 clade were classified as ATUE5. To examine the diversity within the ATUE5 lineage, we constructed a phylogeny using 53 modern and 43 historical ATUE5 genomes. SNPs were identified and filtered as described above, yielding a total of 8,184 biallelic SNPs. A maximum likelihood tree was constructed also using IQ-TREE v2.1.4 (33) applying TVM+F+ASC+R4 as the best substitution model (34).

Ascertainment of tail fiber assembly (*TFA*) and hypothetical tail fiber (*HTF*) haplotypes in historical samples

To capture reads representing the full spectrum of tailocin genetic diversity, we extended the *P. viridiflava* p25.C2 reference genome with six major haplotypes of the highly polymorphic tail fiber (*HTF*) and tail fiber assembly (*TFA*) genes (8). Historical *P. viridiflava* reads were then mapped to this extended reference as described above. Reads mapping to the tailocin cluster between the flanking genes *trpE* and *trpG*, as well as all reads mapping to any haplotype of the *HTF* and *TFA* genes were used for *de novo* assembly using SPAdes v3.15.0 (35). Collinearity between the resulting assemblies and the reference *HTF* and *TFA* genes was assessed using Minimap2 v2.28 (17, 35), and the best-matching *HTF* and *TFA* variants for each assembly were identified by ranking the proportion of coverage obtained from the Minimap2 mapping.

To determine *HTF* haplotypes in historical strains that had insufficient coverage for *de novo* assembly of the *HTF* gene, we performed *k*-mer–based classification of *HTF* sequences. Reference sets of unique *k*-mers (k = 31) were constructed from the seven *HTF* variants (**Fig. S18**). Only *k*-mers exclusive to any of the *HTF* haplotypes and absent from whole-genome backgrounds were retained. To enhance the discriminatory power of our analysis, we filtered *k*-mers across haplotypes to ensure a minimum edit distance of ≥2 between them. The only exception was the haplotype with the smallest *k*-mer set (*HTF*_p23.B8), for which *k*-mers were removed only from the larger overlapping sets. Using this set of filtered *HTF* haplotype *k*-mers, we queried *k*-mer match counts for each historical isolate with Jellyfish v2.2.10 (36) (**Fig. S19**) (all additional *k*-mer profiles are available on GitHub). Additionally, we removed *k*-mers with a fold coverage less than the genome-wide mean minus 0.5 standard deviations. For each isolate, we calculated the proportion of *k*-mers matching each haplotype and assigned the haplotype with the highest proportion as the dominant one (**Table S8**). In cases where multiple haplotypes displayed similar dominant proportions, potential co-infections were recorded and confirmed through inspection of genome-wide *k*-mer profiles (**Fig. S19**). Modern high-quality reads were used as benchmarks to validate the *k*-mer approach and compare it with the local assembly method (**Fig. S14**).

O-antigen biosynthesis cluster (OBC) gene presence/absence analysis

We determined the presence of six O-antigen biosynthesis genes (*wfgD, rmlC_1, tagG_2, tagH_2, spsA,* and *epsE_4*) (8). A gene was considered present if it met

both of the following criteria: (i) a breadth of coverage ≥ 65% and (ii) a mean depth ≥ the genome-wide average minus 0.25 standard deviations of each isolate; otherwise, it was considered absent.

The *epsE_4* locus required additional analysis because of its extended length (~4.5 kb) and high allelic divergence (**Table S8; see GitHub**). For a fraction of modern strains, the mapping to the *P. viridiflava* p25.C2 *epsE_4* reference resulted in the full recovery of the *epsE_4* gene. In other modern strains, we resorted in the previously published *de novo* assemblies (8) to extend from the conserved mapped region on the assembled contig. This process generated a set of diverse modern *epsE_4* variants. For historical isolates lacking whole-genome assemblies, the modern haplotype set was used as a reference for local assembly. Reads mapping to any variant were extracted and assembled locally using SPAdes v3.15.0. (35). Assembled contigs were then aligned against the modern haplotypes with Minimap2 v2.28 (17) to identify the best-matching variant.

For both modern and historical datasets, rescued *epsE_4* sequences were translated into amino acids using ExPASy (37) and EMBOSS Transeq (38). Amino acid sequences were then aligned with Clustal Omega (39). The resulting multiple sequence alignments and allele length distributions are provided here (**Table S8; see GitHub**).

Linkage disequilibrium and recombination analysis

To evaluate the impact of recombination on the association between *HTF* length variants and OBC haplotypes, we quantified genome-wide linkage disequilibrium (LD) and recombination across *Pseudomonas viridiflava* ATUE5. Variant calling was performed on 53 modern ATUE5 genomes using bcftools v1.11 (31), following the same procedure used for phylogenetic reconstruction. A total of 298,977 SNPs were retained and reformatted to include only the genotype (GT) field for LD estimation. Pairwise LD (r^2) was computed using VCFtools v0.1.17 with the --hap-r2 function (40). LD decay was calculated for SNP pairs separated by 0–2 kb using all SNPs, whereas for 2–2,000 kb using a random subset of 2% to reduce computation time. The median r^2 values were calculated after binning SNP pairs by distance (5 bp bins for short-range and 10 kb bins for long-range intervals).

To further quantify genome-wide recombination, the same 53 modern genomes were analyzed using Recophy (41) with the *P. viridiflava* p25.C2 reference genome under default settings. Recophy mixture-model–based approach to estimate whether SNPs across all pairwise genome comparisons arise via mutation (clonal fraction) or recombination (recombining fraction), from which the ratio of recombined to clonal SNPs was calculated.

Plant infection by syringe-infiltration and bacterial population size assessment

Bacteria were grown for 16 h at 28 °C with vigorous agitation (200 rpm). Suspensions were back diluted 1:10 in 5 ml of selective liquid medium and incubated for an additional 3 h. Cells were centrifuged and pellets were washed 3 times with 10 mM $MgSO_4$ (5 ml). Suspensions were adjusted to OD_{600} = 0.1 (10^8 CFU/ml), 0.01 (10^7 CFU/ml) and 0.001 (10^6 CFU/ml). Ten-week-old *Arabidopsis*

thaliana plants of the Eyach 1.5-2 and Col-0 ecotypes, grown under long-day conditions (16 h light, constant 23 °C), were used for the infiltration, selecting two leaves at the same phenological stage from 3 individual plants for each treatment. Using a tip-less 1 ml-syringe, half of each leaf was infiltrated on the abaxial face with either mock solution (10 mM $MgSO_4$) or bacterial suspension (final volume = *ca.* 5-15 µL). Infiltrated plants were returned to the growth chamber and incubated under a transparent plastic lid to maintain high humidity. At two and five days post-infiltration, infiltrated leaves were collected and ground in 1 ml of 10 mM $MgSO_4$. Homogenates were serially diluted (five 1:10 dilutions), and three 10 µL drops of each dilution were spotted onto selective solid medium. After 48 h of static incubation at 28 °C, colonies were counted in each drop to estimate bacterial population size. Significant differences between the WT and O-antigen mutant for each condition were assessed using a Student's t-test, Welch's t-test, or Wilcoxon rank-sum test, as appropriate.

Bacterial growth *in vitro*

Bacteria were grown overnight at 28 °C with vigorous agitation (200 rpm). Cells were centrifuged and overnight pellets were washed three times with PBS. Suspensions were adjusted to OD_{600} = 0.01 in liquid LB. Two hundred microliters of suspension were aliquoted in a 96-well flat transparent plate, considering 4 technical replicates per strain. LB media with no inoculum (sterile) provided the reference for optical density measurements. The plate was placed in a humidity cassette and then loaded into the microplate reader (Spark®, TECAN, Switzerland) where it was continuously shaken and incubated at 28 °C, and optical density at 600 nm was measured every 15 minutes for 24 h. Statistical analysis was performed with three independent biological replicates. Growth curve parameters were extracted using the growthcurver R package and differences were assessed by one-way analysis of variance (ANOVA1) followed by Tukey's post hoc test for each parameter.

Code

Code has been deposited to GitHub:
https://github.com/JiajunCui-jjc/HTF_OBC_historical_analysis.git
https://github.com/talia-backman/Ps1524_tailocin_tradeoffs

Figures

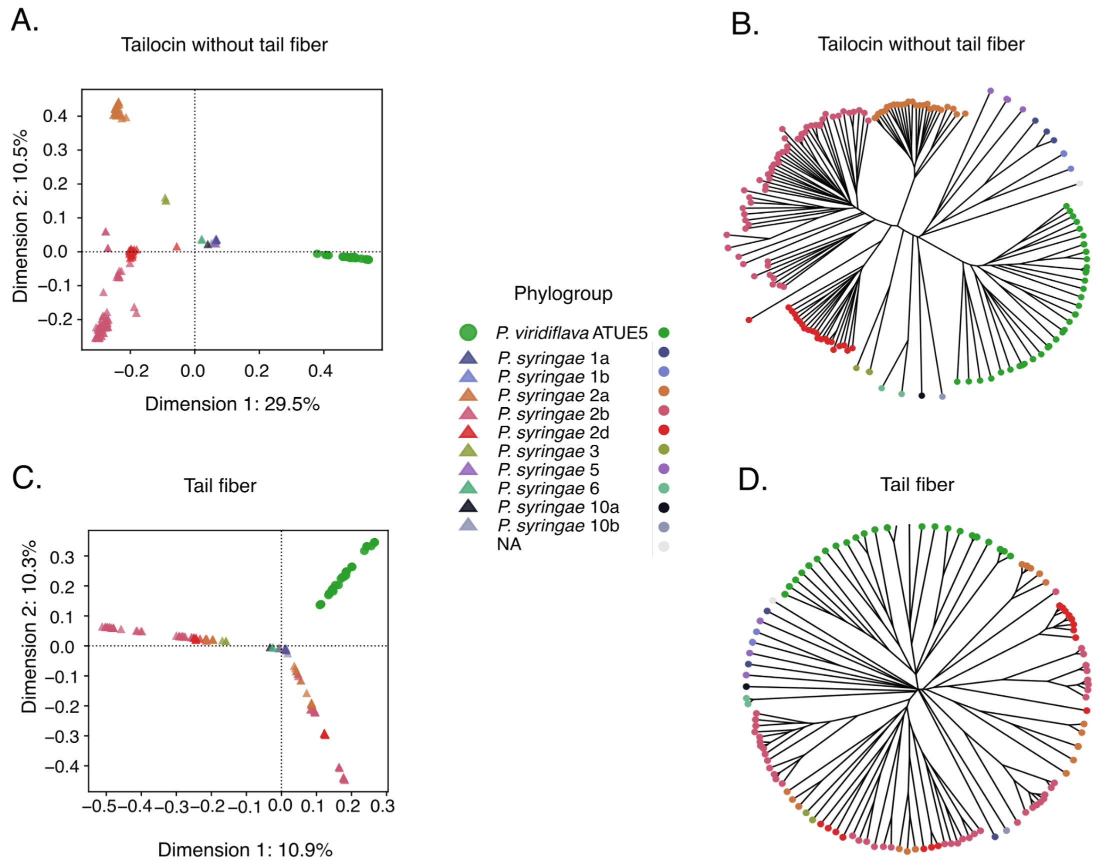

Figure S1: *K*-mer based clustering of tailocin gene islands and tail fibers (*HTF*) of *Pseudomonas viridiflava* ATUE5 and other members of the *Pseudomonas syringae* species complex. A. Principal coordinate analysis (PCoA) of tailocins gene islands (excluding the *HTF*) based on Jaccard distances derived from *k*-mer comparisons. The axes indicate the percentage of the variation explained by each principal coordinate. **B.** Neighbor-joining tree based in the same distances as A of tailocins gene islands (excluding the *HTF*). **C.** Principal coordinate analysis of the *HTF* based on Jaccard distances derived from *k*-mer comparisons (same axes as in A). **D.** Neighbor-joining tree based in the same distances as C of the *HTF*. In both the PCoA and the trees, color and shape denote phylogroup and shape assignments, respectively.

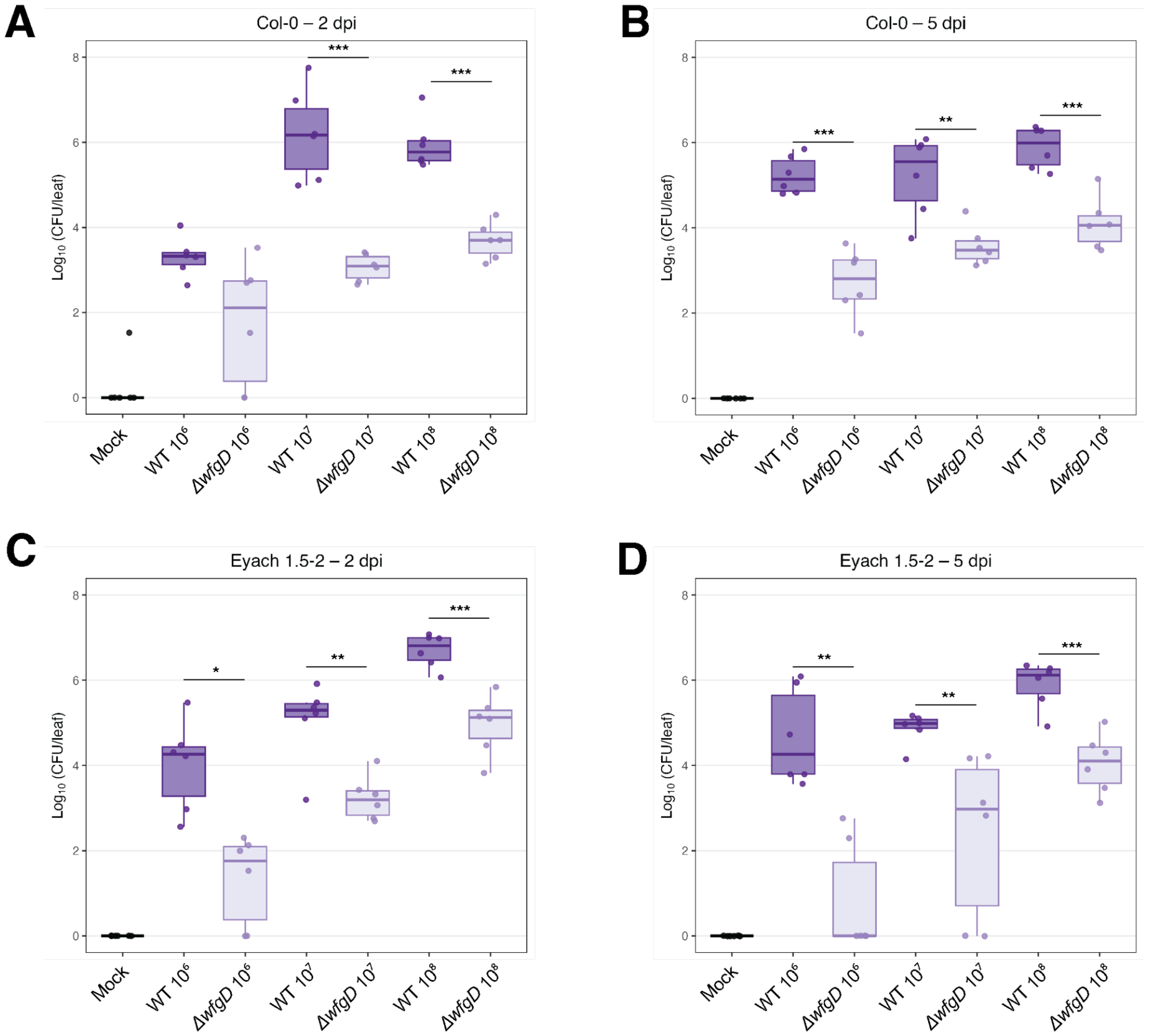

Figure S2: Bacterial population size of p25.C2 WT and O-antigen mutant *ΔwfgD* in Col-0 and Eyach 1.5-2 at 2 and 5 days post-infiltration (dpi) at different inoculum concentrations. The population size of the WT strain was significantly smaller than that of the *ΔwfgD* mutant in all but one condition. Differences between the two strains were assessed using a Student's t-test, Welch's t-test, or Wilcoxon rank-sum test, as appropriate (p-values: *** < 0.001, ** < 0.01, * < 0.05). Data represent 3 technical replicates per condition across 6 biological replicates.

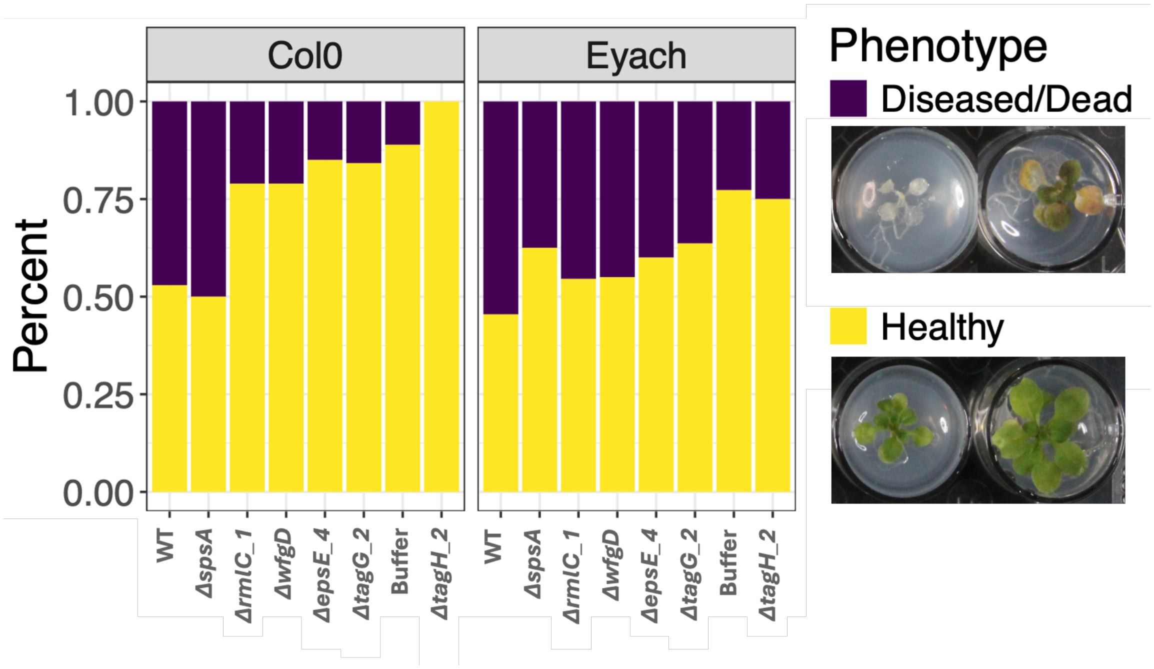

Figure S3: Proportion of plants exhibiting diseased/dead (purple) or healthy (yellow) phenotypes 7 dpi. Across both ecotypes, O-antigen mutants tended to show a higher proportion of healthy plants than WT, although differences were not statistically significant after multiple testing corrections. WT caused disease more frequently but not uniformly, indicating variability in pathogenic outcomes or flood inoculation. Photos of representative diseased, dead, and healthy plants are shown below the legend. Each well is 1.5 cm across.

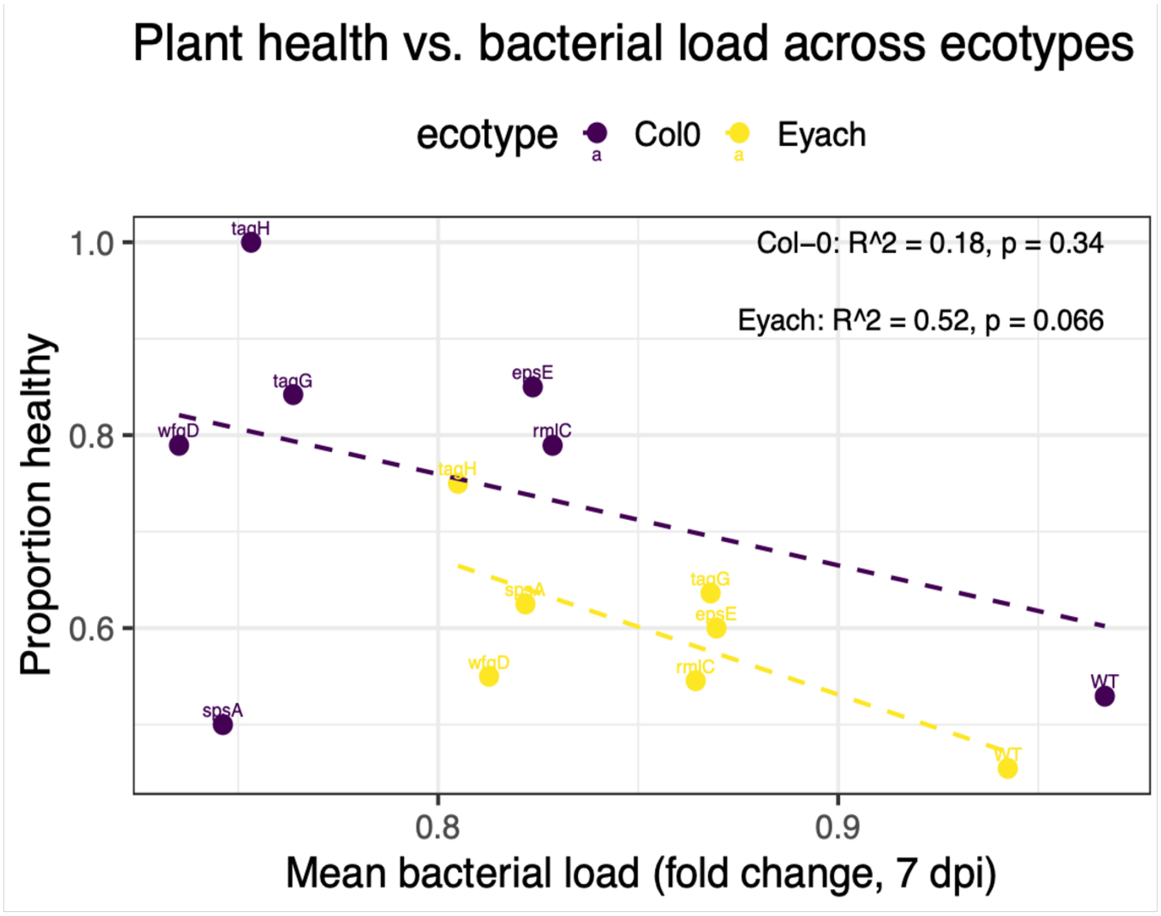

Figure S4: Mean bacterial load (x-axis) is negatively associated with plant health (y-axis) across strains. Each point represents a strain's average 7 dpi fold change (from Fig. 4C) and the corresponding proportion of healthy plants (from Fig. S3). Linear regressions are shown for each ecotype. Across all strains, higher bacterial load tended to predict reduced plant health (pooled model $R^2 = 0.45$, $p = 0.037$; slope $p = 0.086$), although this trend was not significant within Col-0 ($R^2 = 0.18$, $p = 0.34$) and only slightly in Eyach 1.5-2 ($R^2 = 0.52$, $p = 0.066$), reflecting background-specific variation in the relationship between colonization and disease severity.

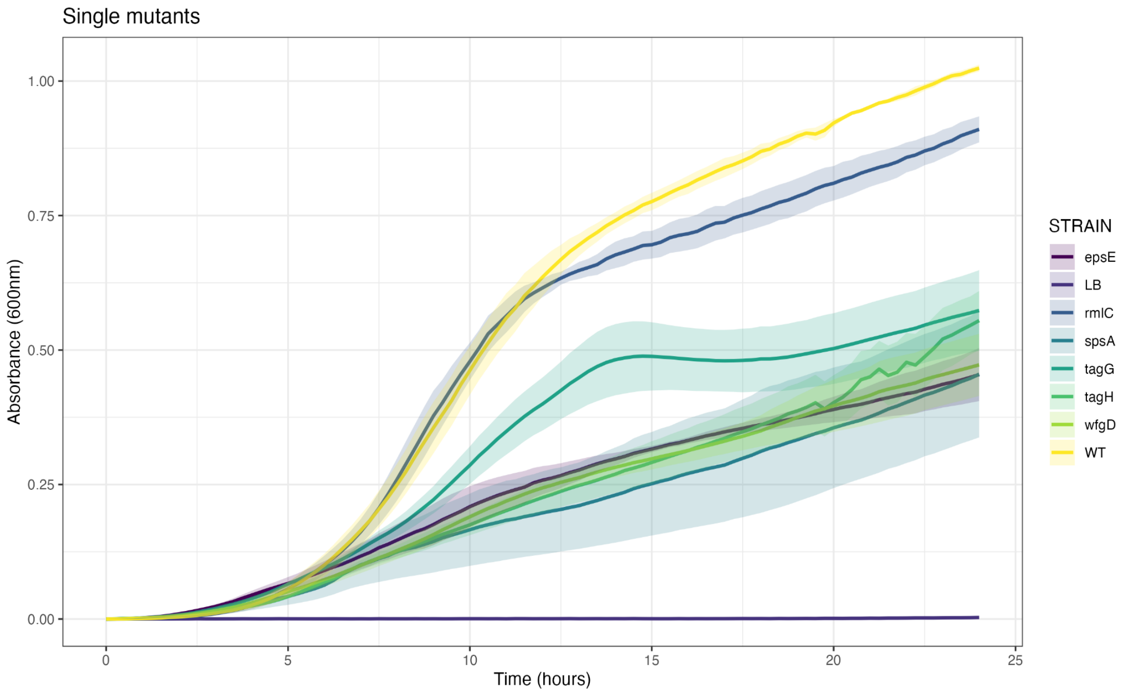

Figure S5: Growth curves *in vitro* of p25.C2 (WT) and the O-antigen mutants. Overnight cultures were adjusted to OD_{600} = 0.01 in rich medium (LB) and absorbance was measured for 24 h. Data represent 4 technical replicates per strain across 3 independent biological replicates. Significant differences among strains are reported in Table S3.

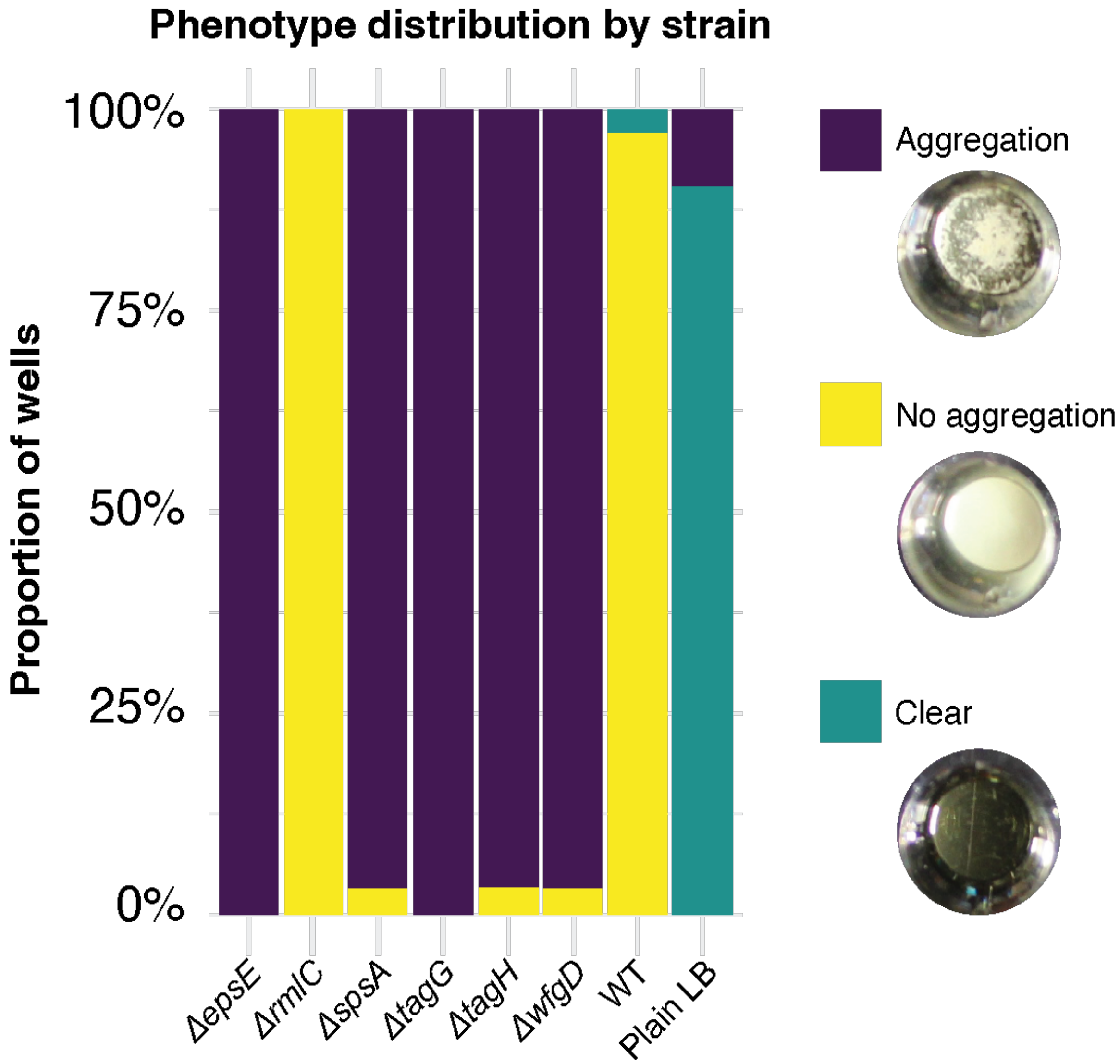

Figure S6: Proportion of wells displaying visible aggregation phenotypes after 48 hours of static incubation at 28°C. Wells were categorized as clear (no growth), growth with no aggregation, or growth with aggregation. All O-antigen mutants except Δ*rmlC* showed significantly higher aggregation frequency than the WT strain (p25.C2; Fisher's exact test, Benjamini–Hochberg-adjusted $p < 10^{-16}$). Data represent 10 technical replicates per strain across 3 biological replicates. Representative photographs illustrate each phenotype category: clear, growth without aggregation, and growth with aggregation.

A

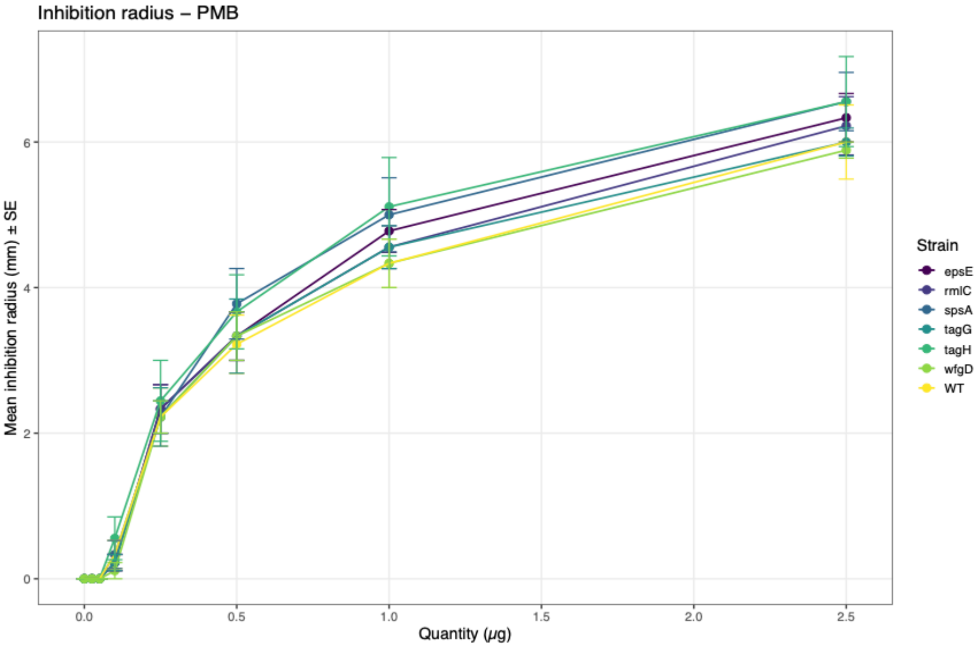

B

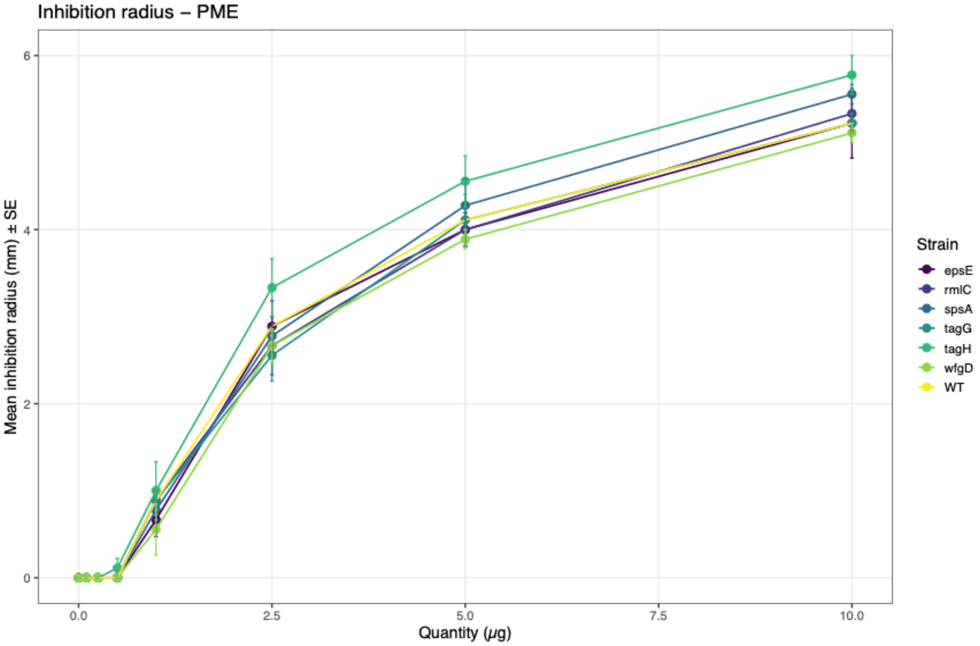

Figure S7: Sensitivity to polymyxin B (A) and polymyxin E (B) in p25.C2 (WT) and the O-antigen mutant. Filter paper disks were saturated with varying quantities of each polymyxin and placed onto freshly streaked bacterial lawns on rich agar medium (LB). Inhibition radii were measured after overnight static incubation at 28 °C. Data represent 3 technical replicates per condition across 3 biological replicates. The absence of significant difference among strains at each compound concentration was assessed using one-way ANOVA or Kruskal–Wallis tests, as appropriate.

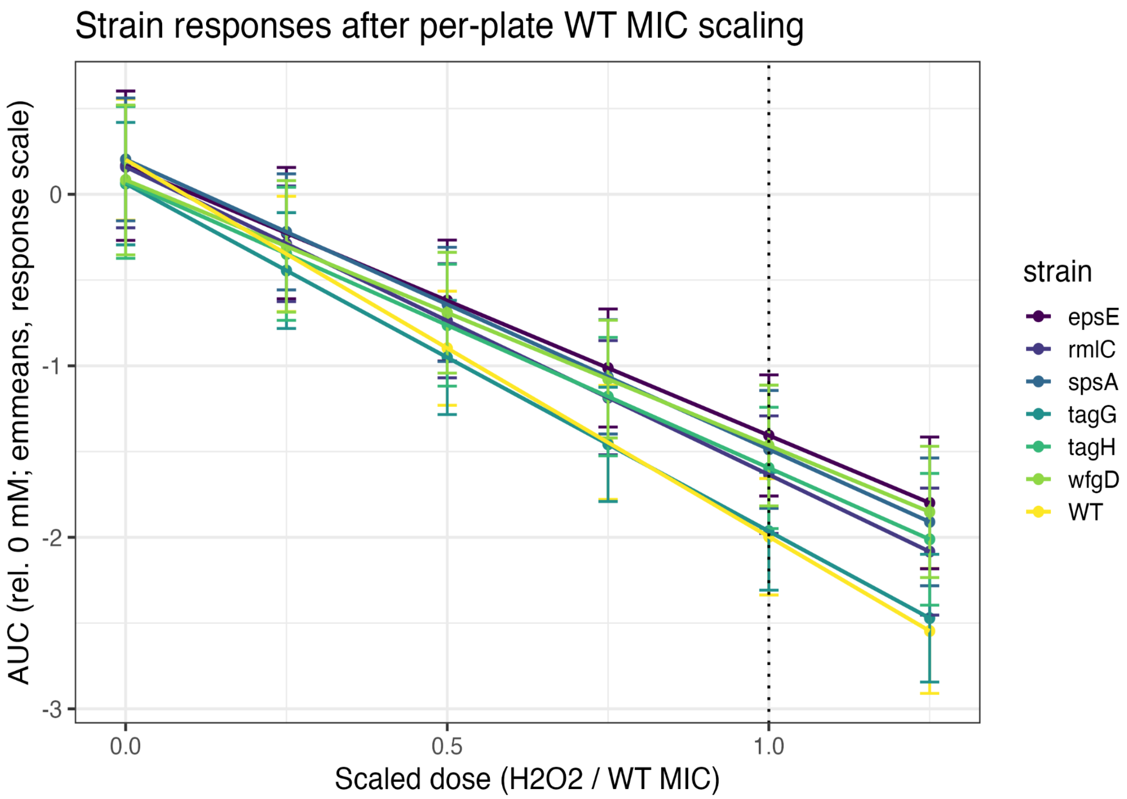

Figure S8: O-antigen mutants and WT exhibit equivalent tolerance to oxidative stress. Area-under-the-curve (AUC) values from growth curves at increasing H_2O_2 concentrations were normalized to 0 mM for each strain and replicate. Each plate's doses were scaled by its WT minimum inhibitory concentration (MIC) to correct for between-experiment variation. Mixed-effects modeling with emmeans contrasts revealed no significant differences between any OPS mutant and WT ($p > 0.05$). Lines show estimated marginal means ± 95% confidence intervals from four replicates.

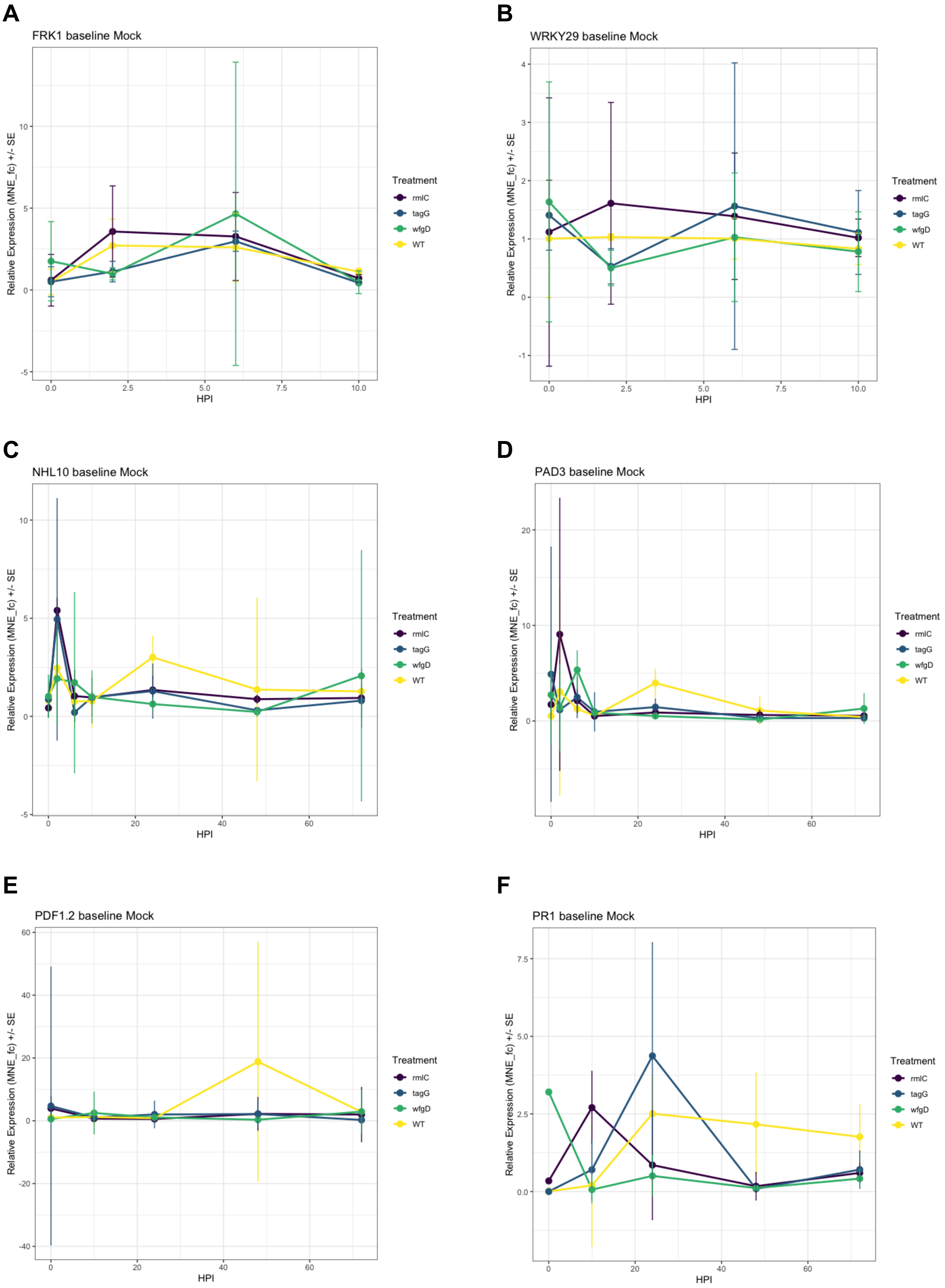
A
FRK1 baseline Mock
B
WRKY29 baseline Mock
C
NHL10 baseline Mock
D
PAD3 baseline Mock
E
PDF1.2 baseline Mock
F
PR1 baseline Mock
Relative Expression (MNE_fc) +/- SE
HPI
Treatment
rmlC
tagG
wfgD
WT

Figure S9: Immunity induction in *Arabidopsis thaliana* Col-0 following infection with p25.C2 (WT) and O-antigen mutants. Plants were flood-inoculated, and expression of immune marker genes *FRK1* (A), *WRKY29* (B), *NHL10* (C), *PAD3* (D), *PDF1.2* (E), and *PR1* (F) was assessed at 0, 2, 6, 10, 24, 48, and 72 hours post-infection (HPI). Expression was normalized to the housekeeping gene *PP2A* and expressed as fold change (fc, relative to mock) of the mean normalized expression (MNE), accounting for PCR efficiency. Data represent three technical replicates per condition across three biological replicates. The absence of a significant difference among strains at each time point was assessed for each target using one-way ANOVA or Kruskal–Wallis tests, as appropriate.

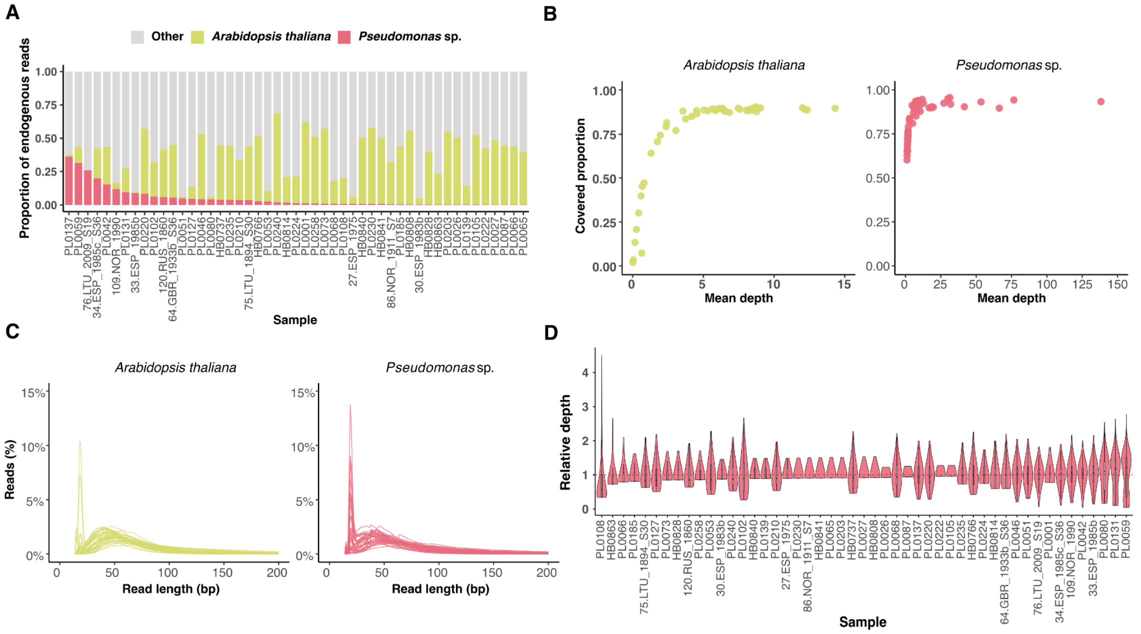

Figure S10: Endogenous DNA profiles reveal variation in historical pathogen representation. A. Endogenous read composition across all 49 historical metagenomic libraries. Stacked bars represent the proportion of reads assigned to *Arabidopsis thaliana* (olive-yellow), *Pseudomonas* sp. (red), and other taxa (grey) within each metagenome. **B.** A scatterplot showing the genome-wide mean read depth (x-axis) vs the breadth of genomic coverage (y-axis) for the *A. thaliana* (olive-yellow) and *Pseudomonas* sp. (red) genomes. Each point represents one historical sample. **C.** Read length distributions of *A. thaliana* (olive-yellow) and *Pseudomonas* sp. (red) reads from 39 historical samples. X-axis was limited to read lengths ≤ 200 bp to provide higher resolution. The remaining ten samples from Lopez et al. 2025 (27) are shown separately in Fig. S11. **D.** Violin plots of *Pseudomonas* sp. whole-genome *k*-mer depth across historical isolates. The x-axis represents individual samples, and the y-axis shows *k*-mer depth normalized to each genome's mean depth (mean = 1). The distributions reveal that most historical infections were dominated by a single *Pseudomonas* strain.

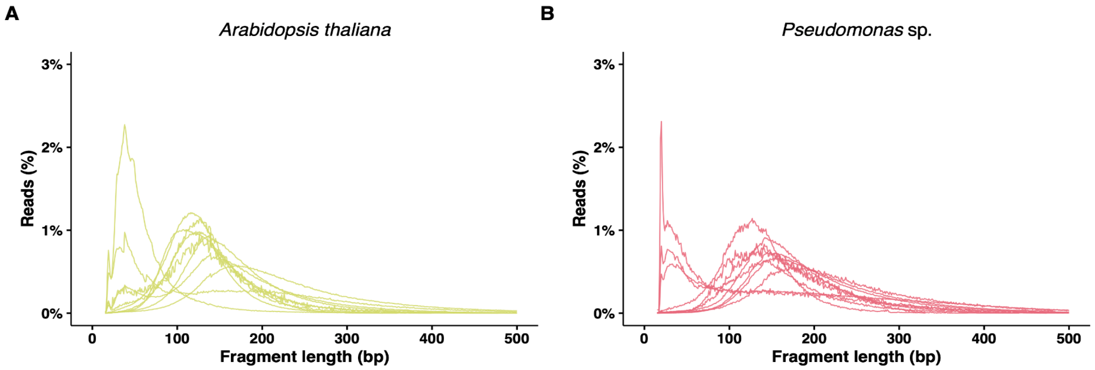

Figure S11: Read length distributions of reads mapped to *Arabidopsis thaliana* (olive-yellow) and *Pseudomonas* sp. (red) for the ten historical metagenomes from Lopez et al. 2025 (27). X-axis was limited to read lengths ≤ 500 bp to provide higher resolution.

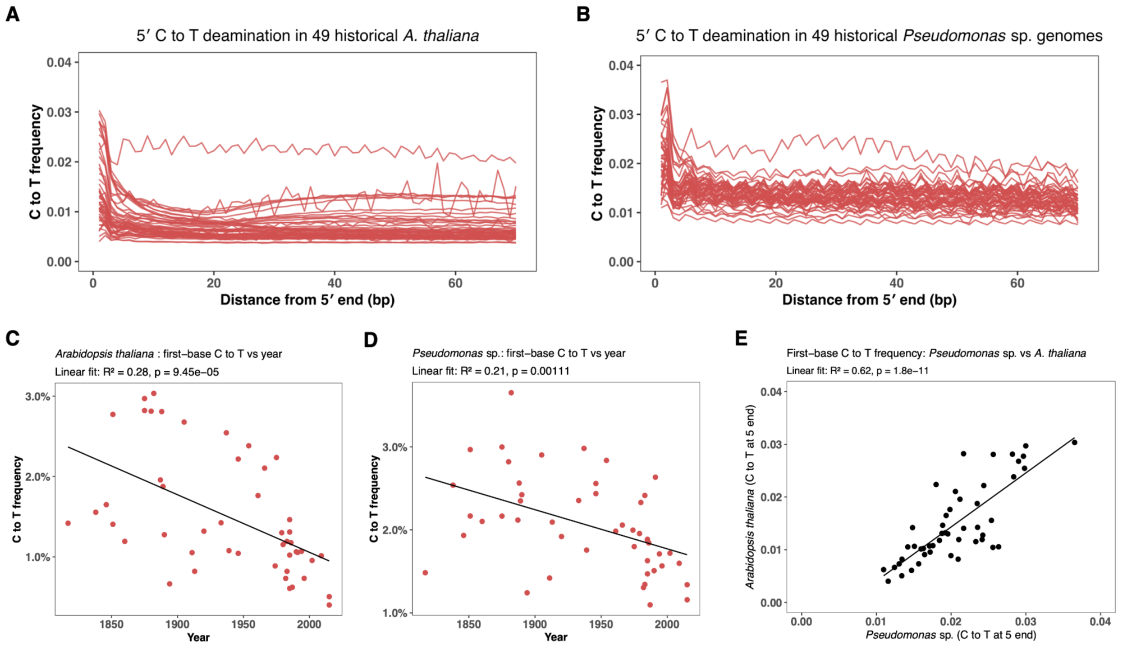

Figure S12: DNA damage patterns in host and pathogen reads support the authenticity of historical metagenomes. Accumulated C-to-T frequencies at the 5′ ends of sequencing reads for 49 *Arabidopsis thaliana* (**A.**) and 49 *Pseudomonas* sp. genomes (**B.**). The y-axis represents C-to-T substitution frequency, and the x-axis shows positions in base pairs from the 5′ end of each read. Both datasets exhibit a typical C-to-T substitutions pattern decaying from the 5′ ends, consistent with historical DNA damage. **C-D.** Scatter plots showing the relationship between the proportion of C-to-T substitutions at the first base (y-axis) and the collection year of each sample (x-axis) for *A. thaliana* (**C.**) and *Pseudomonas* sp. (**D.**). Linear regressions reveal significant negative correlations (*A. thaliana*: R^2=0.28, p value=9.45 x 10^{-5}; *Pseudomonas* sp.: R^2=0.21, p value=1.11 x 10^{-3}), indicating that older samples exhibit higher levels of cytosine deamination. Sample PL0087 was excluded due to missing year information. **E.** Correlation between C-to-T substitution frequencies at the 5′ first bases of reads mapped to *Pseudomonas* sp. vs *A. thaliana*. A significant positive correlation confirms that DNA damage signatures are acquired at similar rates between hosts and pathogens.

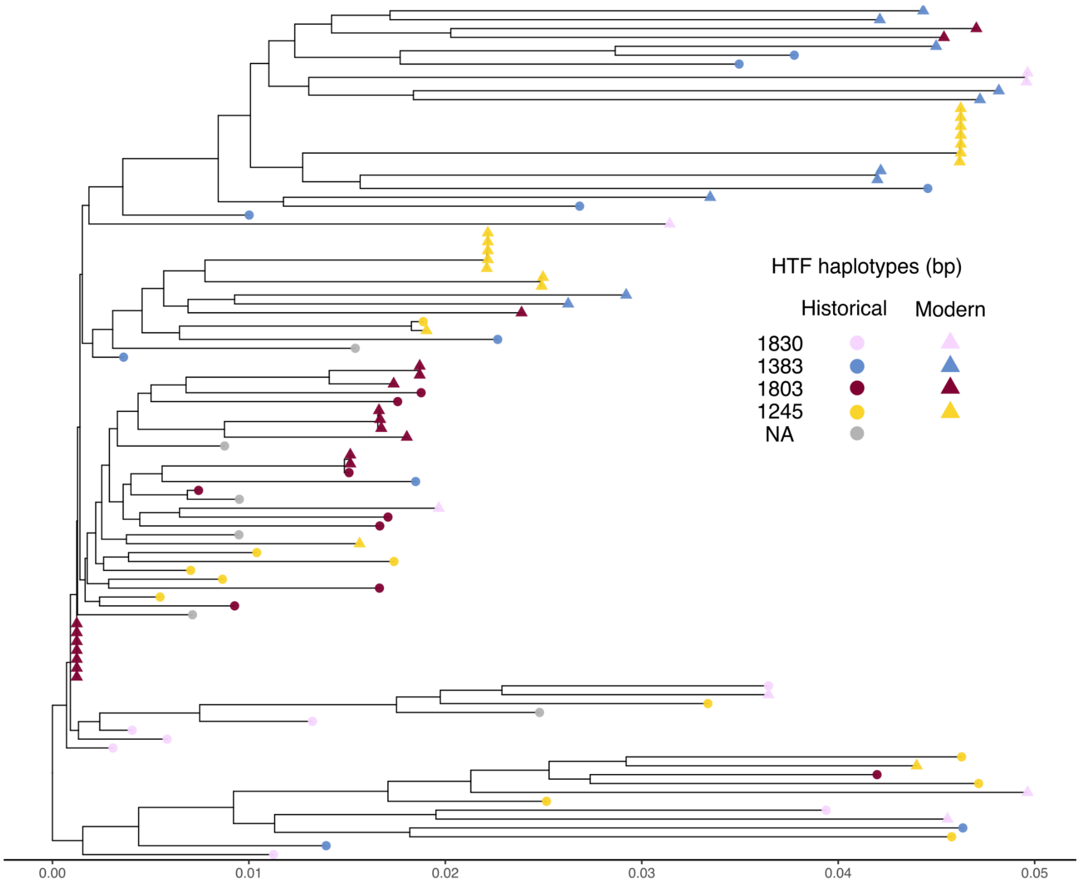

Figure S13: Historical and modern ATUE5 reveal phylogenetic continuity over centuries. Midpoint-rooted maximum-likelihood tree of 43 historical (circles) and 53 modern (triangles) *Pseudomonas viridiflava* ATUE5 genomes. Tip colors indicate the four dominant *HTF* length variants: 1830 bp (pink), 1383 bp (blue), 1803 bp (red) and 1245 bp (yellow). Historical *HTF* variants are broadly distributed across all major branches of the ATUE5 phylogeny, indicating long-term temporal continuity within the lineage.

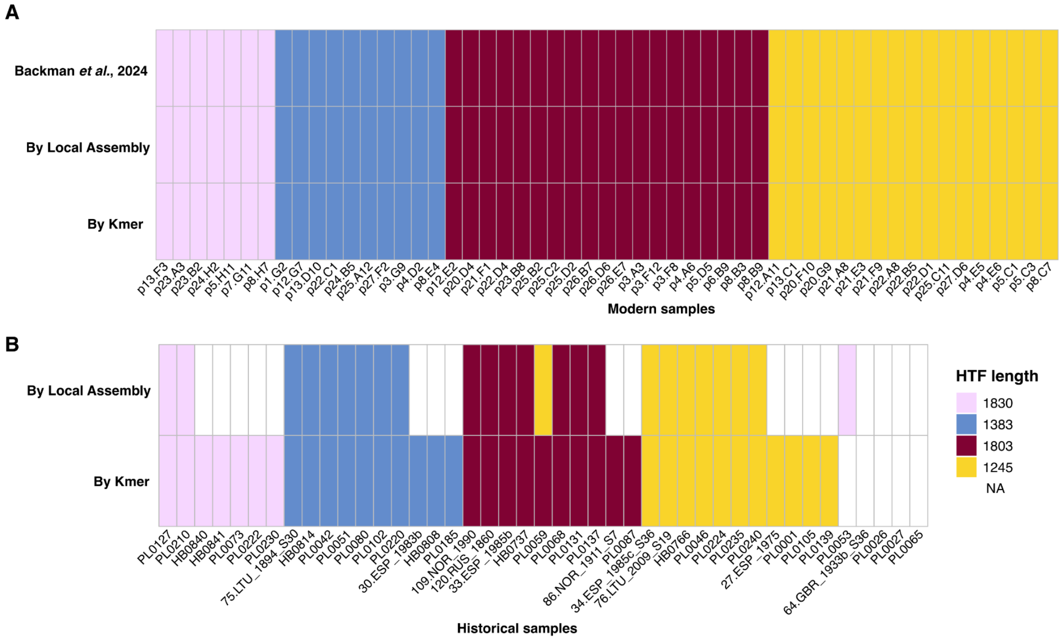

Figure S14: High concordance between *k*-mer and local assembly methods validates *HTF* haplotype reconstructions in historical genomes. A. All 53 modern genomes yielded fully concordant haplotype calls across both methods, consistent with previous assignments (8), which validated the robustness of our approach. **B.** Of 43 historical genomes, 25 haplotypes were assigned by local assembly, and 38 by the *k*-mer method. Concordance among haplotypes assigned by both methods was 96% (23 of 24), with one sample (PL0059) showing conflicting haplotypes (1245 bp vs. 1803 bp), likely due to coinfection (Fig. S19). We used the 23 concordant and 15 additional *k*-mer–derived haplotypes for downstream analyses.

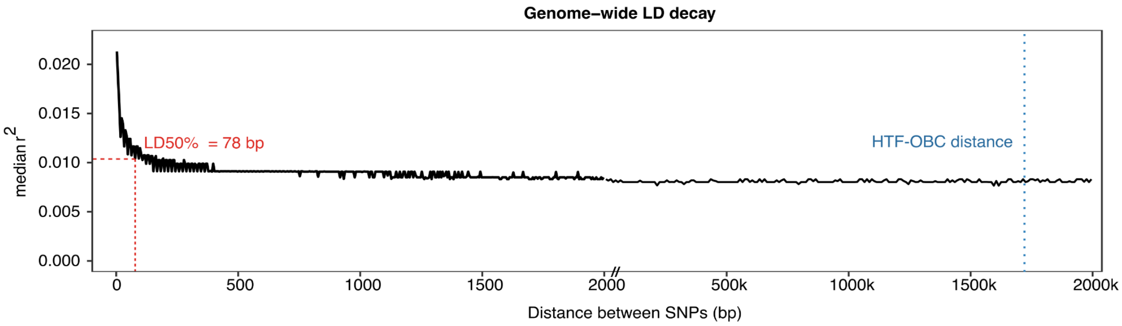

Figure S15: Genome-wide LD decay in *Pseudomonas viridiflava* ATUE5. Pairwise linkage disequilibrium (LD; median r²) between biallelic SNPs was calculated across 53 modern ATUE5 genomes and plotted against SNP distance. LD was estimated for SNP pairs spanning 0–2 kb using all SNPs, and for 2–2,000 kb using a random 2% subset to reduce computation time. LD decays to 50% of its maximum value at 78 bp (LD50; red dashed lines) and reaches background levels by < 2 kb. The genetic distance between the *HTF* and OBC loci (1.7 Mb; blue dashed line) is shown for reference.

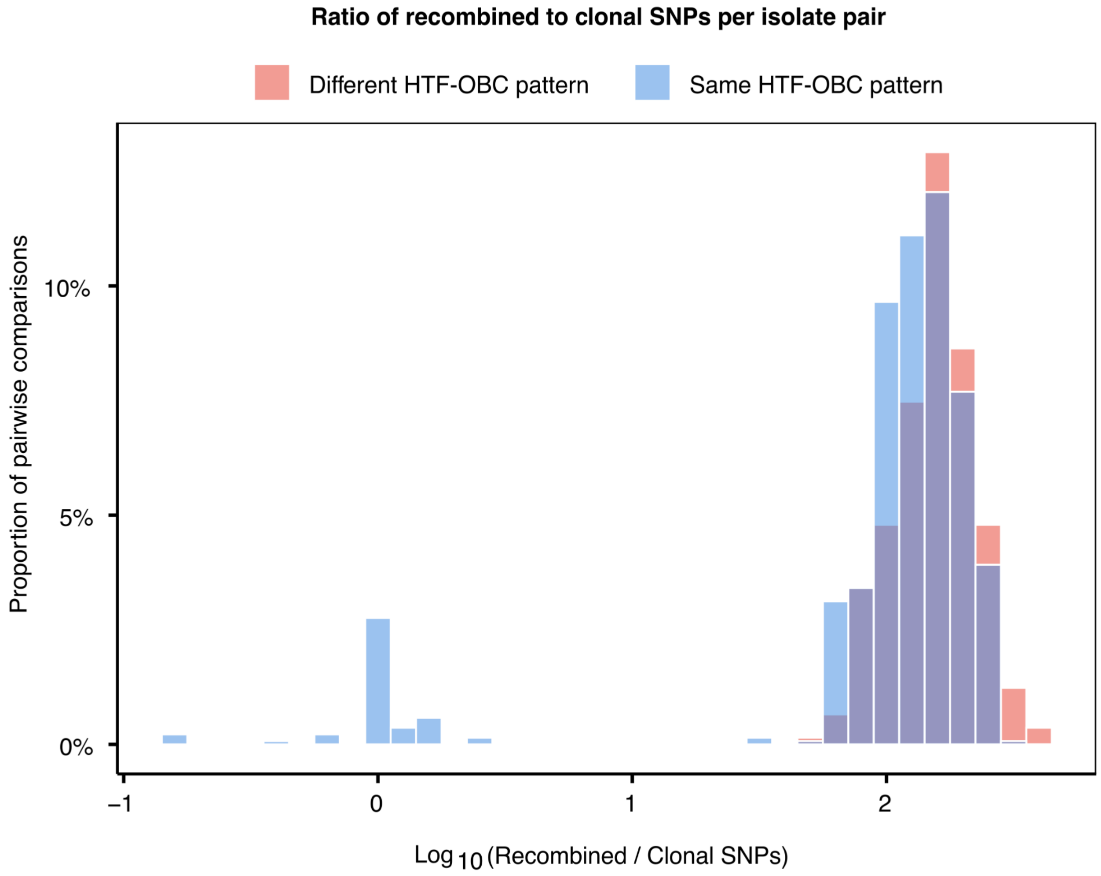

Figure S16: Ratio of recombined to clonal SNPs in pairwise comparisons of *Pseudomonas viridiflava* ATUE5. Distribution of $\log_{10}$(Recombined/Clonal SNPs) across 1,378 pairwise comparisons among 53 modern ATUE5 genomes. Bars indicate the proportion of pairs sharing the same (blue) or different (red) *HTF*–OBC pattern. The purple color indicates the overlap of the two proportions for each bin. 1,318 pairs (95.6%) exhibited $\log_{10}$(Recombined / Clonal SNPs) > 1, indicating that recombination generated more than tenfold the number of clonal SNPs. On average, recombination accounted for 96% of SNPs per isolate.

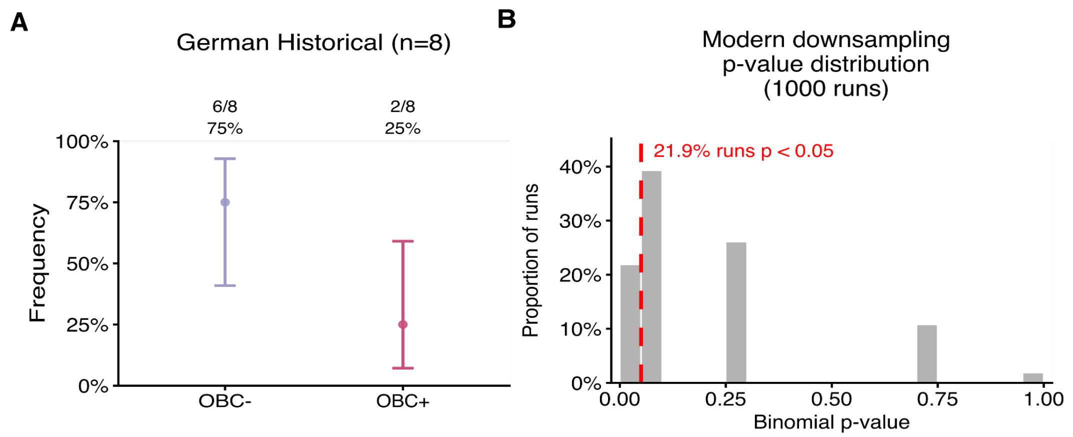

Figure S17: Limited power to detect OBC frequency differences in German historical and downsampled modern *Pseudomonas viridiflava* isolates. A. Observed frequencies of OBC^{-} and OBC^{+} in German historical isolates (n = 8) are shown with Wilson 95% confidence intervals. The deviation from a 50% expectation is not significant (exact binomial test, $p = 0.289$). **B.** To assess the effect of sampling size, modern German isolates (n = 1,312) were repeatedly downsampled to n = 8 without replacement across 1,000 iterations. For each iteration, an exact binomial test was performed to evaluate deviation from a 50% OBC^{-} frequency. The resulting p-value distribution shows that only 21.9% of replicates yield $p < 0.05$ (red dashed line), with a median p-value of 0.07, indicating limited power and that small samples do not reliably capture the true modern OBC frequency.

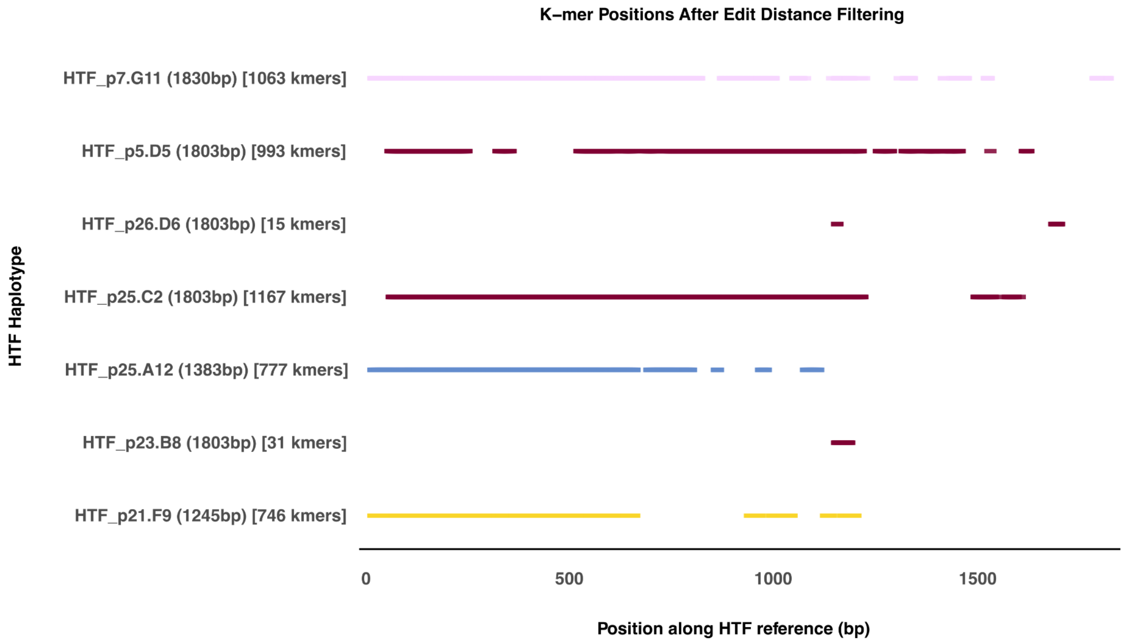

Figure S18: Distribution of diagnostic *k*-mers across *HTF* reference sequences. Each tick represents the position of a *k*-mer along the *HTF* nucleotide sequences (x-axis) after applying an edit-distance filter (>=2). Y-axis labels show *HTF* haplotype names, and the total number of retained *k*-mers per haplotype.

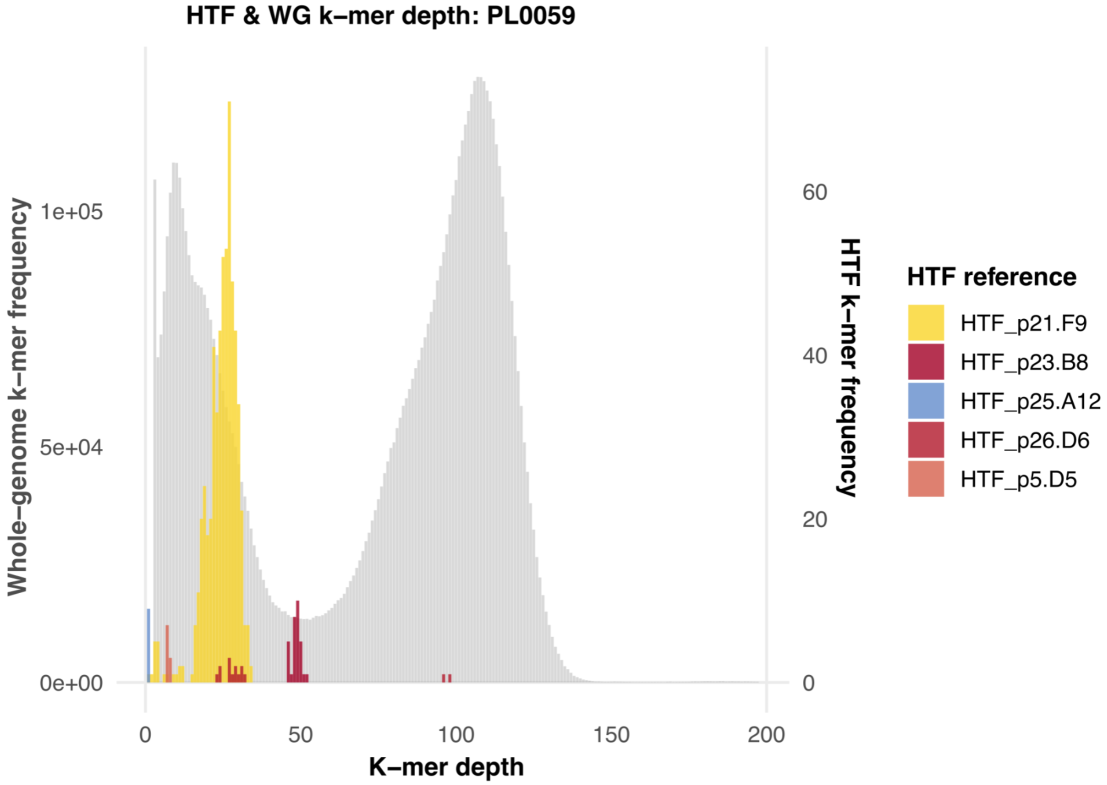

Figure S19: Bimodal *k*-mer depth distribution of PL0059 suggests coinfection. The histograms represent *k*-mer depth distributions. The grey background shows the whole-genome distribution, and the colored histograms represent the distribution for each of the diagnostic *HTF* haplotype *k*-mers. The bimodal pattern in the whole genome *k*-mer distribution indicates potential coinfection in PL0059. *K*-mer distributions for other isolates are available on GitHub.

Tables

Table S1: RB-TnSeq significant resistance trade-off genes.

Barcode ID	Log_2 (fold change) in Col-0	p-value Col-0	Log (fold change) after tailocin treatment	p-value tailocin assay	Log_2 (fold change) in Eyach1.5-2	p-value Eyach 1.5-2	Name gene or product	Significance in both ecotypes
BJEIHDPM_02277	-2.284	1.09E-06	3.560	1.38E-15	-1.570	7.8E-04	*chpB*	Both
BJEIHDPM_03520	-0.790	0.023	0.633	0.035	0.140	0.685	*yciF*	Col-0 only
BJEIHDPM_02879	-1.281	0.023	2.396	1.69E-09	-0.365	0.512	BolA-like protein	Col-0 only
BJEIHDPM_04292	-0.092	0.759	3.436	7.25E-16	-0.710	0.025	Hypothetical protein	Eyach 1.5-2 only
BJEIHDPM_01314	-1.152	0.024	5.499	5.78E-33	-1.479	0.005	Glycosyltransferase	Both
BJEIHDPM_01216	0.083	0.826	1.199	1.46E-06	-0.907	0.021	*ureC_2*	Eyach 1.5-2 only
BJEIHDPM_00728	-0.775	0.018	0.704	0.006	0.155	0.638	*ugpC_1*	Col-0 only
BJEIHDPM_03096	-0.709	0.022	0.530	0.030	0.063	0.838	*cmoB*	Col-0 only
BJEIHDPM_03578	-0.807	0.006	1.719	8.36E-15	-0.900	0.003	*flgE*	Both
BJEIHDPM_00926	-0.964	0.035	5.653	3.2E-29	-0.731	0.117	*rmlC_1*	Col-0 only
BJEIHDPM_00922	-0.379	0.026	6.969	3.02E-41	-0.355	0.040	*rfbB*	Both
BJEIHDPM_00925	-1.200	2E-09	4.909	2.38E-29	-1.363	2.09E-11	*wfgD*	Both
BJEIHDPM_00927	-2.879	7.87E-13	4.696	8.7E-23	-2.469	1.27E-09	*tagG_2*	Both
BJEIHDPM_00929	-0.747	7.4E-04	4.991	1.01E-34	-1.107	8.12E-07	*epsE_4*	Both
BJEIHDPM_00928	-3.293	5.98E-14	4.377	8.78E-20	-3.892	1.87E-16	*tagH_2*	Both
BJEIHDPM_00924	-1.212	8.23E-06	6.776	5.76E-40	-1.092	7.84E-05	*rmlA2_1*	Both
BJEIHDPM_00535	-0.200	0.526	2.040	1.74E-17	-0.909	0.005	*dapL*	Eyach 1.5-2 only
BJEIHDPM_03394	-1.276	1.2E-04	5.187	2.43E-28	-1.436	2.27E-05	*tagO*	Both
BJEIHDPM_03395	-1.018	2.79E-07	5.024	3.51E-29	-0.955	2E-06	*pglF*	Both
BJEIHDPM_01313	-1.324	1.3E-09	4.865	7.59E-30	-0.934	2.32E-05	O-antigen ligase domain-containing protein	Both
BJEIHDPM_01315	-2.185	1.55E-06	6.460	2.76E-33	-2.926	1.52E-09	Glycosyltransferase	Both

Table S2: Comparative glycosyl composition analysis of p25.C2 (WT) LPS and the O-antigen mutants. The LPS was analyzed by the GC-MS after acidic methanolysis, and conversion of the monosaccharides to trimethylsilyl (TMS)-methyl glycosides.

Strain	*ΔwfgD*	*ΔrmlC*	*ΔtagG*	*ΔtagH*	*ΔepsE*	*ΔspsA*	WT
Sugar residue							
Rha	42.8	92.2	69.4	60.6	33.2	24.7	94.7
Glc	27.9	1.0	10.1	8.3	28.2	8.3	0.7
Hep*	0.0	0.0	0.0	0.0	0.0	0.0	0.0
Kdo	27.6	5.9	19.4	29.3	36.3	63.9	2.4
FucNAc**	0.2	0.6	0.1	0.0	0.0	0.0	2.0
QuiNAc**	tr.	tr.	tr.	tr.	tr.	0.0	tr.
GalNAc	0.1	tr.	0.1	0.1	0.1	0.2	0.0
GlcNAc	1.4	0.3	0.9	1.7	2.2	2.9	0.1
	Mol %						

Legend: tr.- below 0.1%; . Rha- rhamnose; ; FucNAc- N-acetyl-fucosamine; Glc-glucose; Kdo- 3-deoxy-D-manno-octulosonic acid; GalNAc- N-acetyl galactosamine, Hep- heptose; GlcNAc- N-acetyl glucosamine; *- the detection of heptose in the applied hydrolytic condition (1 M HCL-MeOH) was not possible due to the phosphorylation of the Hep residue in the LPS; **- due to unavailability of authentic standards of FucNAc, Ara4N, QuiNAc, we used the relative response factor of GlcNAc to calculate the relative mol %.

Table S3: Statistics associated with the growth curves of p25.C2 WT versus O-antigen mutants.

PARAMETER	STRAIN	WT	ΔepsE	ΔrmlC	ΔspsA	ΔtagG	ΔtagH	ΔwfgD
k	WT							
	ΔepsE	**						
	ΔrmlC	NS	*					
	ΔspsA	**	NS	NS				
	ΔtagG	**	NS	NS	NS			
	ΔtagH	NS	NS	NS	NS	NS		
	ΔwfgD	**	NS	*	NS	NS	NS	
r	WT							
	ΔepsE	NS						
	ΔrmlC	NS	NS					
	ΔspsA	*	NS	**				
	ΔtagG	NS	NS	NS	*			
	ΔtagH	NS	NS	**	NS	*		
	ΔwfgD	NS	NS	*	NS	NS	NS	
**t_gen**	WT							
	ΔepsE	NS						
	ΔrmlC	NS	NS					
	ΔspsA	*	NS	*				
	ΔtagG	NS	NS	NS	*			
	ΔtagH	NS	NS	*	NS	*		
	ΔwfgD	NS	NS	NS	NS	NS	NS	
**AUC**	WT							
	ΔepsE	***						
	ΔrmlC	NS	**					
	ΔspsA	***	NS	***				
	ΔtagG	**	NS	*	NS			
	ΔtagH	***	NS	**	NS	NS		
	ΔwfgD	***	NS	**	NS	NS	NS	

Growth curve parameters *k*, *r* (growth rates), t_gen (generation time) and AUC (area under the curve) were extracted for each curve (Fig. S5). Statistical analysis was performed on the means of three independent biological replicates with 4 technical replicates each. Differences were assessed by one-way analysis of variance (ANOVA1) followed by Tukey's post hoc test for each parameter (p-values: *** < 0.001, ** < 0.01, * < 0.05).

Table S4: Hydrogen peroxide minimum inhibitory concentrations (MICs) for WT and O-antigen mutants.

Strain	MIC (mM, mean ± SD)	MIC/WT (est.)	p-value
**WT**	5.0 ± 0.6	1.00	–
Δ***epsE***	6.9 ± 2.7	1.26	0.48
Δ***rmlC***	5.3 ± 0.6	1.06	0.17
Δ***spsA***	6.4 ± 2.7	1.26	0.55
Δ***tagG***	4.3 ± 0.6	0.87	0.27
Δ***tagH***	5.2 ± 0.6	1.03	0.87
Δ***wfgD***	6.6 ± 4.3	1.18	0.72

MICs were determined as the lowest concentration of hydrogen peroxide that inhibited growth after 24 h in liquid culture, based on OD_{600} measurements collected in triplicate using a Tecan plate reader. Values represent mean ± SD (mM). “MIC/WT (est.)” indicates the relative MIC compared to wild-type; p-values reflect pairwise t-tests between each mutant and WT.

Table S5: Plasmids used in this study for Gateway cloning.

Product	Sequence	Additional information
p25.C2 Δ*wfgD* attB DNA fragment	5'-GGGGACAAGTTTGTACAAAAAAGCAGGCTAACAGCGCCAAGGCTGGAAAGTGGCCTGCCTCGAAGAAATCGCGTACACCAATGGCTGGATCACACCAGAACAACTCGGCGCGCAAGCGCAGAAGCTGAAAAAAACCGGTTACGGTCAGTACCTGCAAAAACTGCTGGATCTGGGATCGTTCTGATTTCCGGCGCCTCTTTACACTTCATACCGCAAGGCGATGGACAGCTGACACGCTCATCCGCCTCCGTGGCATGAAAGCGCCTAGCCAGTACAAGCCATTGATAAATAAGGTCAATGCCTGCGCGAGAAGATTCACGACCGCCAGCTAAGCCCTGAACAGAATTGTCGGGAACGAGCAAACACCTAGCGATCCTATGGTTAAATTCCCGCCATCGAATGTTCTCCGAAGGCCGCCTATGTTAATGTACCCAGCTTTCTTGTACAAAGTGGTCCCC-3'	
p25.C2 Δ*rmlC* attB DNA fragment	5'-GGGGACAAGTTTGTACAAAAAAGCAGGCTATGACTCCCGCTCTCCACGCCGTGCAGAAGCCTTTCGCCGGAATCGTTCGTCTGCGCTTCCATCGCGCATGTCATACCTCTTCAAAGTGCTTTTTCCATATGCCCTCACACGTCGGGCAGCAAGCATCCATTATCAGACGCTTTTCCAAACCAGTGCCGCACGAGTCTGCGCTGGTCAGTTTTTCGGAGATACCCTACGTATGAATCCCCATATGACGCCATCTGCCGGCCCCTTGAGACTGGTGCACAGCCTCTGGACGAACCGCTCGTTGATTTCACAGATGACCCGACGCGAAGTCATCGGTCGGTATCGCGGCTCCGTCATGGGGCTGGCCTGGTCATTCTTCAACCCGGTTCTGATGCTGGCGGTATACACCTTCGTCTTTTCCGAAATTTTCAGTACCCAGCTTTCTTGTACAAAGTGGTCCCC-3'	
p25.C2 Δ*tagG* attB DNA fragment	5'-GGGGACAAGTTTGTACAAAAAAGCAGGCTTTCGCCCATGGCTTCGTGGTGTTATCCGAAAGCGCCGAGTTCCTGTACAAGACCACGGATTACTGGGCGCCAGAGCATGAACGCAGTCTGGCCTGGAACGATTCGAGCGTAGCTATCCAATGGCCCATGAGCGAGCAGCCTGCGCTGTCCGCCAAGGATCAGAACGCTGTGCGTTTCGAAGACGCCGAGGTGTTCGCTTAAATGTCATCTGATCAACCGGTTATCAGCGTAAACAACGTCTCCAAGTGTTTTTACACCTACGACAAGCCCCATGAGCGTCTCAAACAGGCCCTGGTGCCGAGACTGCAGCGTTGGGCAGGCAAACAGGCCACTACCACGTACGGTAAAGAGTTCTGGGCGCTGCGAGACATCAGCTTCCATGTCAACAAAGGCGAGACTGTGGGCATTGTCGACCCAGCTTTCTTGTACAAAGTGGTCCCC-3'	

p25.C2 Δ*tagH*	pTB041	Ordered from TWIST
p25.C2 Δ*epsE_4* attB<br><br>DNA fragment	5'-GGGGACAAGTTTGTACAAAAAAGCAGGCTAAGGCATGCCTGAAATTCAAGAGCTCTTTGGCAACCGGCGATTACTTCATATCCGTCGGGATTGCTTCCCGAGAAGGTGAAGAAATCGTCCCTCATGACAGACGCTACGACTCCATACATTTCGTCGTAGAGCCCACCCCCAACCTCCTTGGTTTGATCGACCTGGGTGCGTCCATGGATATCGAACCGGTAACAGTTAATGCTTGAACATGAATATATTCCCCATATCCATTATGCCAAATACGAATAGCAAAGGTATGCAATGAACGTAGTGCAGAGACGTCCCGAAAAAATCGTCACGATGCTAAGGCTGATACTTATACTATTACCGGTCGTGAGCTTCTTAATAAGCGTATGGTCATGGCTGAGGTACGGCGTTGACATTCCAGTTTATGATGACTGGCGTCACCCAGCTTTCTTGTACAAAGTGGTCCCC-3'	
p25.C2 Δ*spsA*	pTB043	Ordered from TWIST
Flanking arm screening<br><br>amplification	• p25.C2ΔwfgD F: 5'-AACAGCGCCAAGGCTGGAAA-3', R: 3'-ACATTAACATAGGCGGCCTTCGG-5',<br>• p25.C2ΔrmlC_1 F: 5'-ATGACTCCCGCTCTCCACGC-3', R: 3'-ACTGAAAATTTCGGAAAAGACGAAGGTGT-5',<br>• p25.C2ΔtagG_2 F: 5'-TTCGCCCATGGCTTCGTGG-3', R: 3'-CGACAATGCCCACAGTCTCGC-5',<br>• p25.C2ΔtagH_2 F: 5'-TCTATCCAATCGCCGCGCTG-3', R: 3'-ACGTCAGGTGATAAAGCTTTGGCC-5',<br>• p25.C2ΔepsE_4 F: 5'-AAAGGCATGCCTGAAATTCAAGAGCT-3', R: 3'-GACGCCAGTCATCATAAACTGGAATGT-5',<br>• p25.C2ΔspsA F: 5'-TCCCTTACAGTGGAACATTCGCG-3', R: 3'-ACACACAGACCGGCACGTG-5'	wfgD WT= 1309bp<br>rmlC WT= 946bp<br>tagG WT = 1228bp<br>tagH WT = 1772bp<br>epsE WT= 5227bp<br>spsA WT= 1374bp
sacB screening<br><br>amplification	F: 5'-tgatttctccggtaaacattacggc-3', R: 3'-cctctgtttgtcatatagcttgtaatcac-5'	762bp
tetR screening<br><br>amplification	F: 5'-accctggatgctgtaggcatagg-3', R: 3'-gccggaagcgagaagaatcataatg-5'	897bp

Table S6: qPCR primers and amplification program used in this study.

Gene	Sequence 5'-3'		Amplicon length
PP2A	FOR: TAACGTGGCCAAAATGATGC<br>REV: GTTCTCCACAACCGCTTGGT		61 bp
FRK1	FOR: TTCGGATTCGGCGTTTGTTG<br>REV: GCTTCTCTTGCTTTGAGGGA		118 bp
WRKY29	FOR: AGAATCTGTTGTCGGACGCA<br>REV: TGCCAAACACCCTTTTGAGC		113 bp
NHL10	FOR: ATCGAAGCTCATGCCTACTACG<br>REV: TTGGCTTCTCGCACAACTTC		113 bp
PAD3	FOR: TTGTGCTAAAGGCTGAAGCG<br>REV: ACCGGAGATACAGTCGATGAAC		92 bp
PDF1.2	FOR: ACCCTTATCTTCGCTGCTCTTG<br>REV: TTGGCTTCTCGCACAACTTC		85 bp
PR1	FOR: CTCATACACTCTGGTGGG<br>REV: TTGGCACATCCGAGTC		191 bp
**AMPLIFICATION PROGRAM**			
	**40 X**		**Up to 95 °C**
95 °C	95 °C	62 °C	62 °C + 0.3 °C/cycle
3'	30"	30"	30"

Table S7: Historical sample metadata.

SAMPLE	TYPE	YEAR	COUNTRY	At_proportion	At_covered_proportion	At_average_depth	Ps_proportion	Ps_covered_proportion	Ps_average_depth	GROUP	HERBARIUM*	SOURCE
PL0001	Plant tissue herbarium	1966	Spain	61.00%	87.61%	8.76	1.31%	90.66%	4.58	ATUE5	MGC	This study. ENA: PRJEB98841
PL0026	Plant tissue herbarium	2002	Sweden	50.00%	89.86%	12.01	0.50%	72.29%	1.58	ATUE5	OHN	This study. ENA: PRJEB98841
PL0059	Plant tissue herbarium	1985	Spain	12.10%	87.14%	6.85	31.47%	92.87%	137.41	ATUE5	RJB	This study. ENA: PRJEB98841
PL0073	Plant tissue herbarium	1991	Spain	56.30%	88.14%	8.13	1.05%	83.24%	2.83	ATUE5	RJB	This study. ENA: PRJEB98841
PL0087	Plant tissue herbarium	NA	Spain	44.00%	88.46%	8.37	0.33%	62.29%	1.13	ATUE5	RJB	This study. ENA: PRJEB98841
PL0102	Plant tissue herbarium	1888	Germany	25.60%	89.59%	7.46	6.34%	95.09%	31.13	ATUE5	TUE	This study. ENA: PRJEB98841
PL0105	Plant tissue herbarium	1920	Sweden	51.90%	89.42%	8.6	0.48%	64.37%	1.18	ATUE5	LND	This study. ENA: PRJEB98841
PL0108	Plant tissue herbarium	1946	Sweden	19.60%	89.74%	9.05	0.82%	80.50%	4.89	nonATUE5	LND	This study. ENA: PRJEB98841
PL0131	Plant tissue herbarium	1980	Spain	18.00%	87.70%	6.55	9.59%	92.67%	53.07	ATUE5	RJB	This study. ENA: PRJEB98841
PL0139	Plant tissue herbarium	1996	Sweden	13.90%	79.11%	2.36	0.48%	70.92%	1.52	ATUE5	OHN	This study. ENA: PRJEB98841
PL0185	Plant tissue herbarium	1994	Germany	43.20%	89.06%	8.35	0.60%	81.74%	2.7	ATUE5	STG	This study. ENA: PRJEB98841
PL0203	Plant tissue herbarium	1880	United Kingdom	54.80%	88.49%	8.06	0.50%	72.39%	1.68	nonATUE5	NHM	This study. ENA: PRJEB98841
PL0210	Plant tissue herbarium	1838	United Kingdom	30.30%	84.96%	4.18	3.70%	87.36%	10.36	ATUE5	NHM	This study. ENA: PRJEB98841
PL0220	Plant tissue herbarium	1946	United Kingdom	48.60%	88.47%	5.84	8.37%	89.32%	18.44	ATUE5	NHM	This study. ENA: PRJEB98841
PL0222	Plant tissue herbarium	1887	United Kingdom	42.10%	89.12%	8.55	0.36%	64.92%	1.23	ATUE5	NHM	This study. ENA: PRJEB98841
PL0224	Plant tissue herbarium	1889	United Kingdom	20.20%	89.01%	6.4	1.36%	93.00%	7.01	ATUE5	NHM	This study. ENA: PRJEB98841

PL0230	Plant tissue herbarium	1851	United Kingdom	57.30%	90.32%	8.68	0.73%	78.60%	2.01	ATUE5	NHM	This study. ENA: PRJEB98841
PL0235	Plant tissue herbarium	1905	United Kingdom	40.30%	86.39%	4.54	3.90%	91.81%	8.56	ATUE5	NHM	This study. ENA: PRJEB98841
PL0240	Plant tissue herbarium	1875	United Kingdom	66.70%	88.97%	7.57	2.02%	84.63%	7.38	ATUE5	NHM	This study. ENA: PRJEB98841
PL0258	Plant tissue herbarium	1913	United Kingdom	49.90%	88.39%	6.42	1.15%	83.22%	2.75	nonATUE5	NHM	This study. ENA: PRJEB98841
PL0027	Plant tissue herbarium	1939	Sweden	48.30%	89.78%	8.85	0.33%	65.78%	1.27	ATUE5	OHN	This study. ENA: PRJEB98841
PL0042	Plant tissue herbarium	1982	Spain	28.20%	88.62%	8.49	15.37%	93.77%	76.14	ATUE5	RJB	This study. ENA: PRJEB98841
PL0046	Plant tissue herbarium	1974	Spain	49.00%	88.93%	12.17	4.31%	89.76%	19.52	ATUE5	RJB	This study. ENA: PRJEB98841
PL0051	Plant tissue herbarium	1983	Spain	1.50%	20.73%	0.26	4.89%	89.92%	18.44	ATUE5	RJB	This study. ENA: PRJEB98841
PL0053	Plant tissue herbarium	1987	Spain	7.70%	64.01%	1.3	2.68%	94.16%	11.72	ATUE5	RJB	This study. ENA: PRJEB98841
PL0065	Plant tissue herbarium	2015	Spain	39.90%	88.61%	12.35	0.21%	68.88%	1.35	ATUE5	RJB	This study. ENA: PRJEB98841
PL0066	Plant tissue herbarium	2015	Spain	43.40%	89.49%	14.32	0.32%	70.50%	1.61	nonATUE5	RJB	This study. ENA: PRJEB98841
PL0068	Plant tissue herbarium	1986	Spain	17.20%	89.28%	8.43	1.04%	91.81%	12.58	ATUE5	RJB	This study. ENA: PRJEB98841
PL0080	Plant tissue herbarium	1985	Spain	1.90%	39.76%	0.58	4.23%	94.48%	29.67	ATUE5	RJB	This study. ENA: PRJEB98841
PL0127	Plant tissue herbarium	1979	Spain	9.10%	45.22%	0.68	4.61%	93.15%	8.63	ATUE5	RJB	This study. ENA: PRJEB98841
PL0137	Plant tissue herbarium	1961	Sweden	1.30%	1.89%	0.02	36.32%	89.29%	16.67	ATUE5	OHN	This study. ENA: PRJEB98841
27.ESP_1975	Plant tissue herbarium	1975	Spain	5.50%	7.22%	0.64	0.76%	75.00%	1.71	ATUE5	RJB	(Lopez et al., 2025)
30.ESP_1983b	Plant tissue herbarium	1983	Spain	4.70%	47.14%	0.81	0.54%	75.39%	1.74	ATUE5	RJB	(Lopez et al., 2025)
33.ESP_1985b	Plant tissue herbarium	1985	Spain	0.80%	13.26%	0.16	8.86%	91.39%	31.62	ATUE5	RJB	(Lopez et al., 2025)
34.ESP_1985c_S36	Plant tissue herbarium	1985	Spain	22.30%	81.36%	2.4	19.98%	89.98%	41.23	ATUE5	RJB	(Lopez et al., 2025)
64.GBR_1933b_S36	Plant tissue herbarium	1933	UK	39.50%	74.18%	1.95	5.71%	86.42%	5.51	ATUE5	KEW	(Lopez et al., 2025)

75.LTU_1894_S30	Plant tissue herbarium	1894	Lithuania	40.20%	87.96%	3.56	3.67%	90.64%	6.43	ATUE5	KBI	(Lopez et al., 2025)
76.LTU_2009_S19	Plant tissue herbarium	2009	Lithuania	0.20%	3.36%	0.04	26.02%	89.20%	65.73	ATUE5	RJB	(Lopez et al., 2025)
86.NOR_1911_S7	Plant tissue herbarium	1911	Norway	31.60%	83.44%	3.75	0.60%	67.85%	1.35	ATUE5	UOS	(Lopez et al., 2025)
109.NOR_1990	Plant tissue herbarium	1990	Norway	4.80%	30.04%	0.42	11.96%	91.97%	26.84	ATUE5	UOS	(Lopez et al., 2025)
120.RUS_1860	Plant tissue herbarium	1860	Russia	35.70%	70.54%	1.75	5.96%	89.46%	5.59	ATUE5	KBI	(Lopez et al., 2025)
HB0737	Plant tissue herbarium	1937	Germany	40.80%	88.15%	4.52	3.95%	90.08%	9.5	ATUE5	TUE	(Lang et al., 2024)
HB0766	Plant tissue herbarium	1846	Germany	48.90%	88.31%	5.87	2.85%	88.03%	8.16	ATUE5	STG	(Lang et al., 2024)
HB0808	Plant tissue herbarium	1817	Germany	55.10%	88.06%	5.33	0.56%	59.81%	1.06	ATUE5	STG	(Lang et al., 2024)
HB0814	Plant tissue herbarium	1882	Germany	19.20%	88.51%	5.05	1.82%	93.44%	8.98	ATUE5	STG	(Lang et al., 2024)
HB0828	Plant tissue herbarium	1890	Germany	39.10%	89.47%	6.34	0.52%	73.53%	1.74	nonATUE5	STG	(Lang et al., 2024)
HB0840	Plant tissue herbarium	1851	Germany	49.70%	89.45%	6.09	0.73%	76.28%	1.78	ATUE5	STG	(Lang et al., 2024)
HB0841	Plant tissue herbarium	1875	Germany	49.40%	89.21%	5.68	0.71%	73.19%	1.59	ATUE5	STG	(Lang et al., 2024)
HB0863	Plant tissue herbarium	1954	Somalia	23.20%	76.88%	3.05	0.51%	65.01%	1.49	nonATUE5	STG	(Lang et al., 2024)

Table S8: Summary of tailocin coverage, *HTF* haplotype assignment, and O-antigen presence in historical samples.

SAMPLE	Tailocin_cov_prop	Tailocin_depth	*HTF*_haplotype_bykmer	*HTF*_haplotype_bylocalassembly	*HTF*group_Oantigen_PA	wfgD_PA	rmlC_1_PA	tagG_2_PA	tagH_2_PA	spsA_PA	epsE_4_PA	epsE_4_length
109.NOR_1990	0.9662	28.26	*HTF*_p5.D5 (1803)	*HTF*_p5.D5 (1803)	OBC_present	1	1	1	1	1	1	4665
120.RUS_1860	0.9371	5.91	*HTF*_p5.D5 (1803)	*HTF*_p5.D5 (1803)	OBC_present	0	1	1	1	1	1	4665
27.ESP_1975	0.7961	1.88	*HTF*_p21.F9 (1245)	NA	OBC_present	1	1	1	1	1	NA	NA
30.ESP_1983b	0.8016	1.99	*HTF* _p25.A12 (1383)	NA	OBC_absent	0	0	0	0	0	0	0
33.ESP_1985b	0.9636	34.78	*HTF*_p23.B8 (1803)	*HTF*_p26.D6 (1803)	OBC_present	1	1	1	1	1	1	4827
34.ESP_1985c_S36	0.8867	42.57	*HTF*_p21.F9 (1245)	*HTF*_p21.F9 (1245)	OBC_present	1	1	1	1	1	1	4665
64.GBR_1933b_S36	0.8298	6.03	NA	NA	NA	0	0	0	0	0	0	0
75.LTU_1894_S30	0.9479	7.24	*HTF*_p25.A12 (1383)	*HTF*_p25.A12 (1383)	OBC_absent	0	0	0	0	0	0	0
76.LTU_2009_S19	0.8864	65.25	*HTF*_p21.F9 (1245)	*HTF*_p21.F9 (1245)	OBC_present	1	1	1	1	1	1	4665
86.NOR_1911_S7	0.7649	1.66	*HTF*_p23.B8 (1803)	NA	OBC_present	1	0	1	1	1	NA	NA
HB0737	0.9488	12.32	*HTF*_p23.B8 (1803)	*HTF*_p26.D6 (1803)	OBC_present	1	1	1	1	1	1	4827
HB0766	0.8935	8.33	*HTF*_p21.F9 (1245)	*HTF*_p21.F9 (1245)	OBC_present	1	1	1	1	1	1	4665
HB0808	0.6299	1.24	*HTF*_p25.A12 (1383)	NA	OBC_absent	0	0	0	0	0	0	0
HB0814	0.9414	10.22	*HTF*_p25.A12 (1383)	*HTF*_p25.A12 (1383)	OBC_absent	0	0	0	0	0	0	0
HB0840	0.8095	1.76	*HTF*_p7.G11 (1830)	NA	OBC_absent	0	0	0	0	0	0	0
HB0841	0.7612	1.69	*HTF*_p7.G11 (1830)	NA	OBC_absent	0	0	0	0	0	0	0
p1.G2	0.9472	46.84	*HTF*_p25.A12 (1383)	*HTF*_p25.A12 (1383)	OBC_absent	0	0	0	0	0	0	0
p12.A11	0.9066	42.92	*HTF*_p21.F9 (1245)	*HTF*_p21.F9 (1245)	OBC_present	1	1	0	1	1	1	4827
p12.E2	0.9965	119.05	*HTF*_p25.C2 (1803)	*HTF*_p26.D6 (1803)	OBC_present	1	1	1	1	1	1	4827
p12.G7	0.9472	25.53	*HTF*_p25.A12 (1383)	*HTF*_p25.A12 (1383)	OBC_absent	0	0	0	0	0	0	0
p13.C1	0.908	80.79	*HTF*_p21.F9 (1245)	*HTF*_p21.F9 (1245)	OBC_present	1	1	1	1	1	1	4665
p13.D10	0.9421	82.2	*HTF*_p25.A12 (1383)	*HTF*_p25.A12 (1383)	OBC_absent	0	0	0	0	0	0	0
p13.F3	0.9032	149.3	*HTF*_p7.G11 (1830)	*HTF*_p7.G11 (1830)	OBC_absent	0	0	0	0	0	0	0
p20.D4	0.985	27.7	*HTF*_p25.C2 (1803)	*HTF*_p26.D6 (1803)	OBC_present	1	1	1	1	1	1	4830
p20.F10	0.8983	25.66	*HTF*_p21.F9 (1245)	*HTF*_p21.F9 (1245)	OBC_present	1	1	0	1	1	1	4827
p20.G9	0.8983	23.34	*HTF*_p21.F9 (1245)	*HTF*_p21.F9 (1245)	OBC_present	1	1	0	1	1	1	4827
p21.A8	0.899	47.84	*HTF*_p21.F9 (1245)	*HTF*_p21.F9 (1245)	OBC_present	1	1	0	1	1	1	4827
p21.E3	0.8894	36.2	*HTF*_p21.F9 (1245)	*HTF*_p21.F9 (1245)	OBC_present	1	1	1	1	1	1	4665
p21.F1	0.9455	55.85	*HTF*_p5.D5 (1803)	*HTF*_p26.D6 (1803)	OBC_present	1	1	1	1	1	1	4665
p21.F9	0.915	40.89	*HTF*_p21.F9 (1245)	*HTF*_p21.F9 (1245)	OBC_present	1	1	0	1	1	1	4827
p22.A8	0.8847	35.76	*HTF*_p21.F9 (1245)	*HTF*_p21.F9 (1245)	OBC_present	1	1	1	1	1	1	4665
p22.B5	0.9002	27.86	*HTF*_p21.F9 (1245)	*HTF*_p21.F9 (1245)	OBC_present	1	1	1	1	1	1	4665
p22.C1	0.9312	40.68	*HTF*_p25.A12 (1383)	*HTF*_p25.A12 (1383)	OBC_absent	0	0	0	0	0	0	0
p22.D1	0.8985	9.41	*HTF*_p21.F9 (1245)	*HTF*_p21.F9 (1245)	OBC_present	1	1	1	1	1	1	4740
p22.D4	0.9494	7.01	*HTF*_p26.D6 (1803)	*HTF*_p26.D6 (1803)	OBC_present	1	1	1	1	1	1	4827
p23.A3	0.8558	85.83	*HTF*_p7.G11 (1830)	*HTF*_p7.G11 (1830)	OBC_absent	0	0	0	0	0	0	0
p23.B2	0.8543	49.68	*HTF*_p7.G11 (1830)	*HTF*_p7.G11 (1830)	OBC_absent	1	0	0	0	0	0	0
p23.B8	0.9671	26.47	*HTF*_p23.B8 (1803)	*HTF*_p26.D6 (1803)	OBC_present	1	1	1	1	1	1	4827
p24.B5	0.9463	23.21	*HTF*_p25.A12 (1383)	*HTF*_p25.A12 (1383)	OBC_absent	0	0	0	0	0	0	0
p24.H2	0.8291	28.8	*HTF*_p7.G11 (1830)	*HTF*_p7.G11 (1830)	OBC_absent	0	0	0	0	0	0	0
p25.A12	0.8627	6.84	*HTF*_p25.A12 (1383)	*HTF*_p25.A12 (1383)	OBC_absent	1	0	0	0	0	0	0
p25.B2	0.9983	9.65	*HTF*_p25.C2 (1803)	*HTF*_p26.D6 (1803)	OBC_present	1	1	1	1	1	1	4827
p25.C11	0.9102	8.22	*HTF*_p21.F9 (1245)	*HTF*_p21.F9 (1245)	OBC_present	1	1	1	1	0	NA	NA
p25.C2	1	42.26	*HTF*_p25.C2 (1803)	*HTF*_p26.D6 (1803)	OBC_present	1	1	1	1	1	1	4827
p25.D2	1	11.66	*HTF*_p25.C2 (1803)	*HTF*_p26.D6 (1803)	OBC_present	1	1	1	1	1	1	4827
p26.B7	0.9848	18.48	*HTF*_p25.C2 (1803)	*HTF*_p26.D6 (1803)	OBC_present	1	1	1	1	1	1	4773
p26.D6	0.9423	10.85	*HTF*_p26.D6 (1803)	*HTF*_p26.D6 (1803)	OBC_present	1	1	1	1	1	1	4827

p26.E7	0.9665	26.22	*HTF*_p23.B8 (1803)	*HTF*_p26.D6 (1803)	OBC_present	1	1	1	1	1	1	4827
p27.D6	0.8839	56.04	*HTF*_p21.F9 (1245)	*HTF*_p21.F9 (1245)	OBC_present	1	1	1	1	1	1	4665
p27.F2	0.9471	28.34	*HTF*_p25.A12 (1383)	*HTF*_p25.A12 (1383)	OBC_absent	0	0	0	0	0	0	0
p3.A3	1	13.74	*HTF*_p25.C2 (1803)	*HTF*_p26.D6 (1803)	OBC_present	1	1	1	1	1	1	4827
p3.F12	1	67.93	*HTF*_p25.C2 (1803)	*HTF*_p26.D6 (1803)	OBC_present	1	1	1	1	1	1	4827
p3.F8	0.9725	14.7	*HTF*_p26.D6 (1803)	*HTF*_p26.D6 (1803)	OBC_present	1	1	1	1	1	1	4827
p3.G9	0.9641	38.83	*HTF*_p25.A12 (1383)	*HTF*_p25.A12 (1383)	OBC_absent	0	0	0	0	0	0	0
p4.A6	1	12.73	*HTF*_p25.C2 (1803)	*HTF*_p26.D6 (1803)	OBC_present	1	1	1	0	1	1	4827
p4.D2	0.9521	41.7	*HTF*_p25.A12 (1383)	*HTF*_p25.A12 (1383)	OBC_absent	0	0	0	0	0	0	0
p4.E5	0.8841	8.28	*HTF*_p21.F9 (1245)	*HTF*_p21.F9 (1245)	OBC_present	1	1	1	1	1	1	4665
p4.E6	0.8915	24.02	*HTF*_p21.F9 (1245)	*HTF*_p21.F9 (1245)	OBC_present	1	1	1	1	1	1	4665
p5.C1	0.9244	81.52	*HTF*_p21.F9 (1245)	*HTF*_p21.F9 (1245)	OBC_present	1	1	1	1	1	1	4740
p5.C3	0.8853	52.84	*HTF*_p21.F9 (1245)	*HTF*_p21.F9 (1245)	OBC_present	1	1	1	1	1	1	4665
p5.D5	0.9405	51.93	*HTF*_p5.D5 (1803)	*HTF*_p26.D6 (1803)	OBC_present	1	1	1	1	1	1	4665
p5.H11	0.81	20.36	*HTF*_p7.G11 (1830)	*HTF*_p7.G11 (1830)	OBC_absent	0	0	0	0	0	0	0
p6.B9	0.9682	44.69	*HTF*_p26.D6 (1803)	*HTF*_p26.D6 (1803)	OBC_present	1	1	1	1	1	1	4827
p7.G11	0.8663	51.55	*HTF*_p7.G11 (1830)	*HTF*_p7.G11 (1830)	OBC_absent	0	0	0	0	0	0	0
p8.B3	1	81.84	*HTF*_p25.C2 (1803)	*HTF*_p26.D6 (1803)	OBC_present	1	1	1	1	1	1	4827
p8.B9	0.9546	50.02	*HTF*_p5.D5 (1803)	*HTF*_p26.D6 (1803)	OBC_present	1	1	1	1	1	1	4665
p8.C7	0.9228	47.55	*HTF*_p21.F9 (1245)	*HTF*_p21.F9 (1245)	OBC_present	1	1	1	1	1	1	4665
p8.E4	0.8831	33.34	*HTF*_p25.A12 (1383)	*HTF*_p25.A12 (1383)	OBC_absent	1	0	0	0	0	0	0
p8.H7	0.8614	57.78	*HTF*_p7.G11 (1830)	*HTF*_p7.G11 (1830)	OBC_absent	0	0	0	0	0	0	0
PL0001	0.8967	5.13	*HTF*_p21.F9 (1245)	NA	OBC_present	1	1	1	1	1	1	4740
PL0026	0.8111	1.85	NA	NA	NA	1	0	1	1	1	0	0
PL0027	0.6808	1.37	NA	NA	NA	1	1	1	1	1	0	0
PL0042	0.9445	81.1	*HTF*_p25.A12 (1383)	*HTF*_p25.A12 (1383)	OBC_absent	0	0	0	0	0	0	0
PL0046	0.8973	18.89	*HTF*_p21.F9 (1245)	*HTF*_p21.F9 (1245)	OBC_present	1	1	1	1	1	1	4665
PL0051	0.945	19.86	*HTF*_p25.A12 (1383)	*HTF*_p25.A12 (1383)	OBC_absent	0	0	0	0	0	0	0
PL0053	0.903	11.25	NA	*HTF*_p7.G11 (1830)	NA	0	0	0	0	0	1	4740
PL0059	0.9773	147.81	*HTF*_p23.B8 (1803)	*HTF*_p21.F9 (1245)	OBC_present	1	1	1	1	1	1	4827
PL0065	0.7115	1.47	NA	NA	NA	0	0	1	1	1	0	0
PL0068	0.9509	13.96	*HTF*_p23.B8 (1803)	*HTF*_p26.D6 (1803)	OBC_present	1	1	1	1	1	1	4827
PL0073	0.8247	2.85	*HTF*_p7.G11 (1830)	NA	OBC_absent	0	0	0	0	0	0	0
PL0080	0.9335	28.2	*HTF*_p25.A12 (1383)	*HTF*_p25.A12 (1383)	OBC_absent	0	0	0	0	0	0	0
PL0087	0.706	1.36	*HTF*_p5.D5 (1803)	NA	OBC_present	1	1	1	1	0	NA	NA
PL0102	0.9598	35.59	*HTF*_p25.A12 (1383)	*HTF*_p25.A12 (1383)	OBC_absent	0	0	0	0	0	0	0
PL0105	0.6913	1.4	*HTF*_p21.F9 (1245)	NA	OBC_present	0	1	1	1	1	NA	NA
PL0127	0.9203	8.31	*HTF*_p7.G11 (1830)	*HTF*_p7.G11 (1830)	OBC_absent	0	0	0	0	0	0	0
PL0131	0.972	55	*HTF*_p5.D5 (1803)	*HTF*_p5.D5 (1803)	OBC_present	1	1	1	1	1	1	4665
PL0137	0.9291	17.56	*HTF*_p26.D6 (1803)	*HTF*_p26.D6 (1803)	OBC_present	1	1	1	1	1	1	4827
PL0139	0.7523	1.74	*HTF*_p21.F9 (1245)	NA	OBC_present	1	1	0	1	1	NA	NA
PL0185	0.8486	2.92	*HTF*_p25.A12 (1383)	NA	OBC_absent	0	0	0	0	0	0	0
PL0210	0.8408	9.75	*HTF*_p7.G11 (1830)	*HTF*_p7.G11 (1830)	OBC_absent	0	0	0	0	0	0	0
PL0220	0.873	19.29	*HTF*_p25.A12 (1383)	*HTF*_p25.A12 (1383)	OBC_absent	0	0	0	0	0	0	0
PL0222	0.5953	1.1	*HTF*_p7.G11 (1830)	NA	OBC_absent	0	0	0	0	0	0	0
PL0224	0.9083	7.46	*HTF*_p21.F9 (1245)	*HTF*_p21.F9 (1245)	OBC_present	0	1	1	1	1	1	4665
PL0230	0.7933	2.03	*HTF*_p7.G11 (1830)	NA	OBC_absent	1	0	0	0	0	0	0
PL0235	0.9158	9.25	*HTF*_p21.F9 (1245)	*HTF*_p21.F9 (1245)	OBC_present	1	1	0	1	1	1	NA
PL0240	0.8604	8.04	*HTF*_p21.F9 (1245)	*HTF*_p21.F9 (1245)	OBC_present	0	0	0	0	0	0	0

*** Herbarium: KBI** = Komarov Botanical Institute of RAS (Russia); **KEW** = Kew Botanical Garden (United Kingdom); **LND** = Lund University Botanical Museum (Sweden); **MGC** = Herbario de Málaga (Spain); **NHM** = The Natural History Museum (United Kingdom); **OHN** = Biological Museum Oskarshamn (Sweden); **RJB** = Real Jardin Botanico (Spain); **STG** = Staatliches Museum für Naturkunde Stuttgart (Germany); **TUE** = Herbarium Tubingense University of Tübingen (Germany); **UOS** = University of Oslo (Norway).

SI References

1. T. L. Karasov, *et al.*, Arabidopsis thaliana and Pseudomonas Pathogens Exhibit Stable Associations over Evolutionary Timescales. *Cell Host Microbe* **24**, 168–179.e4 (2018).

2. J. Vacheron, C. M. Heiman, C. Keel, Live cell dynamics of production, explosive release and killing activity of phage tail-like weapons for Pseudomonas kin exclusion. *Commun Biol* **4**, 87 (2021).

3. A. Duque-Jaramillo, *et al.*, The genetic and physiological basis of Arabidopsis thaliana tolerance to Pseudomonas viridiflava. *New Phytol.* **240**, 1961–1975 (2023).

4. K. M. Wetmore, *et al.*, Rapid quantification of mutant fitness in diverse bacteria by sequencing randomly bar-coded transposons. *MBio* **6**, e00306–15 (2015).

5. M. I. Love, W. Huber, S. Anders, Moderated estimation of fold change and dispersion for RNA-seq data with DESeq2. *Genome Biol.* **15**, 550 (2014).

6. J. L. Hartley, G. F. Temple, M. A. Brasch, DNA cloning using in vitro site-specific recombination. *Genome Res.* **10**, 1788–1795 (2000).

7. O. Westphal, K. Jann, Bacterial Lipopolysaccharides Extraction Phenol-Water Further Applications Procedure. *Methods Carbohydrate Chemistry* **5**, 83–91 (1965).

8. T. Backman, *et al.*, A phage tail–like bacteriocin suppresses competitors in metapopulations of pathogenic bacteria. *Science* **384**, eado0713 (2024).

9. J. H. Krauss, J. Weckesser, H. Mayer, Electrophoretic analysis of lipopolysaccharides of purple nonsulfur bacteria. *Int. J. Syst. Bacteriol.* **38**, 157–163 (1988).

10. W. S. York, A. G. Darvill, M. McNeil, T. T. Stevenson, P. Albersheim, “Isolation and characterization of plant cell walls and cell wall components” in *Methods in Enzymology*, Methods in enzymology., (Elsevier, 1986), pp. 3–40.

11. U. R. Bhat, H. Mayer, A. Yokota, R. I. Hollingsworth, R. W. Carlson, Occurrence of lipid A variants with 27-hydroxyoctacosanoic acid in lipopolysaccharides from members of the family Rhizobiaceae. *J. Bacteriol.* **173**, 2155–2159 (1991).

12. J. Binesse, H. Lindgren, L. Lindgren, W. Conlan, A. Sjöstedt, Roles of reactive oxygen species-degrading enzymes of Francisella tularensis SCHU S4. *Infect. Immun.* **83**, 2255–2263 (2015).

13. M. W. Pfaffl, A new mathematical model for relative quantification in real-time RT-PCR. *Nucleic Acids Res.* **29**, e45 (2001).

14. P. Simon, Q-Gene: processing quantitative real-time RT-PCR data. *Bioinformatics* **19**, 1439–1440 (2003).

15. G. Bouras, *et al.*, Pharokka: a fast scalable bacteriophage annotation tool. *Bioinformatics* **39** (2023).

16. G. Bouras, *et al.*, Protein structure-informed bacteriophage genome annotation with Phold. *Nucleic Acids Res* **54** (2026).

17. H. Li, Minimap2: pairwise alignment for nucleotide sequences. *Bioinformatics* **34**, 3094–3100 (2018).

18. A. J. Aylward, S. Petrus, A. Mamerto, N. T. Hartwick, T. P. Michael, PanKmer: k-mer-based and reference-free pangenome analysis. *Bioinformatics* **39** (2023).

19. M. Aton, *et al.*, Scikit-bio: a fundamental Python library for biological omic data analysis. *Nat Methods* **23**, 274–276 (2026).

20. P. J. A. Cock, *et al.*, Biopython: freely available Python tools for computational molecular biology and bioinformatics. *Bioinformatics* **25**, 1422–1423 (2009).

21. C. Fautt, K. Hockett, S. Delattre, D. Baltrus, E. Couradeau, Tailocin tail fiber diversity correlates with rfbD variation in the Pseudomonas syringae species complex. *ISME Commun.* **5**, ycaf099 (2025).

22. C. Fautt, E. Couradeau, K. L. Hockett, Naïve Bayes Classifiers and accompanying dataset for Pseudomonas syringae isolate characterization. *Sci Data* **11**, 178 (2024).

23. S. M. Latorre, P. L. M. Lang, H. A. Burbano, R. M. Gutaker, Isolation, library preparation, and bioinformatic analysis of historical and ancient plant DNA. *Curr. Protoc. Plant Biol.* **5**, e20121 (2020).

24. J. D. Kapp, R. E. Green, B. Shapiro, A Fast and Efficient Single-stranded Genomic Library Preparation Method Optimized for Ancient DNA. *J Hered* **112**, 241–249 (2021).

25. R. Nguyen, J. D. Kapp, S. Sacco, S. P. Myers, R. E. Green, A computational approach for positive genetic identification and relatedness detection from low-coverage shotgun sequencing data. *J Hered* **114**, 504–512 (2023).

26. P. L. M. Lang, *et al.*, Century-long timelines of herbarium genomes predict plant stomatal response to climate change. *Nat Ecol Evol* **8**, 1641–1653 (2024).

27. L. Lopez, *et al.*, Museum Genomics Reveals Temporal Genetic Stasis and Global Genetic Diversity in Arabidopsis thaliana. *Mol Ecol* **34**, e70081 (2025).

28. M. Schubert, S. Lindgreen, L. Orlando, AdapterRemoval v2: rapid adapter trimming, identification, and read merging. *BMC Res Notes* **9**, 88 (2016).

29. D. Swarbreck, *et al.*, The Arabidopsis Information Resource (TAIR): gene structure and function annotation. *Nucleic Acids Res* **36**, D1009–14 (2008).

30. H. Li, R. Durbin, Fast and accurate short read alignment with Burrows-Wheeler transform. *Bioinformatics* **25**, 1754–1760 (2009).

31. P. Danecek, *et al.*, Twelve years of SAMtools and BCFtools. *Gigascience* **10**, giab008 (2021).

32. H. Jónsson, A. Ginolhac, M. Schubert, P. L. F. Johnson, L. Orlando, mapDamage2.0: fast approximate Bayesian estimates of ancient DNA damage parameters. *Bioinformatics* **29**, 1682–1684 (2013).

33. B. Q. Minh, *et al.*, IQ-TREE 2: New Models and Efficient Methods for Phylogenetic Inference in the Genomic Era. *Mol Biol Evol* **37**, 1530–1534 (2020).

34. S. Kalyaanamoorthy, B. Q. Minh, T. K. F. Wong, A. von Haeseler, L. S. Jermiin, ModelFinder: fast model selection for accurate phylogenetic estimates. *Nat Methods* **14**, 587–589 (2017).

35. A. Prjibelski, D. Antipov, D. Meleshko, A. Lapidus, A. Korobeynikov, Using SPAdes De Novo Assembler. *Curr. Protoc. Bioinformatics* **70**, e102 (2020).

36. G. Marçais, C. Kingsford, A fast, lock-free approach for efficient parallel counting of occurrences of k-mers. *Bioinformatics* **27**, 764–770 (2011).

37. E. Gasteiger, *et al.*, ExPASy: The proteomics server for in-depth protein knowledge and analysis. *Nucleic Acids Res* **31**, 3784–3788 (2003).

38. P. Rice, I. Longden, A. Bleasby, EMBOSS: the European Molecular Biology Open Software Suite. *Trends Genet* **16**, 276–277 (2000).

39. F. Sievers, *et al.*, Fast, scalable generation of high-quality protein multiple sequence alignments using Clustal Omega. *Mol Syst Biol* **7**, 539 (2011).

40. P. Danecek, *et al.*, The variant call format and VCFtools. *Bioinformatics* **27**, 2156–2158 (2011).

41. T. Sakoparnig, C. Field, E. van Nimwegen, Whole genome phylogenies reflect the distributions of recombination rates for many bacterial species. *Elife* **10** (2021).
